# Effect of Periodate-Induced Cross-linking on Dual Anticancer Drug Release from Poly(2-isopropyl-2-oxazoline)/Tannic Acid-Based Layer-by-Layer Microparticles

**DOI:** 10.1021/acsomega.4c03977

**Published:** 2024-09-11

**Authors:** Esma Ugur, Gökçe Tidim, Dilara Gundogdu, Cemre Alemdar, Goksu Oral, H. Hazal Husnugil, Sreeparna Banerjee, Irem Erel-Goktepe

**Affiliations:** † Department of Chemistry, Middle East Technical University, 06800 Cankaya, Ankara, Türkiye; ‡ Department of Biology, Middle East Technical University, 06800 Cankaya, Ankara, Türkiye; § Center of Excellence in Biomaterials and Tissue Engineering, Middle East Technical University, 06800 Cankaya, Ankara, Türkiye

## Abstract

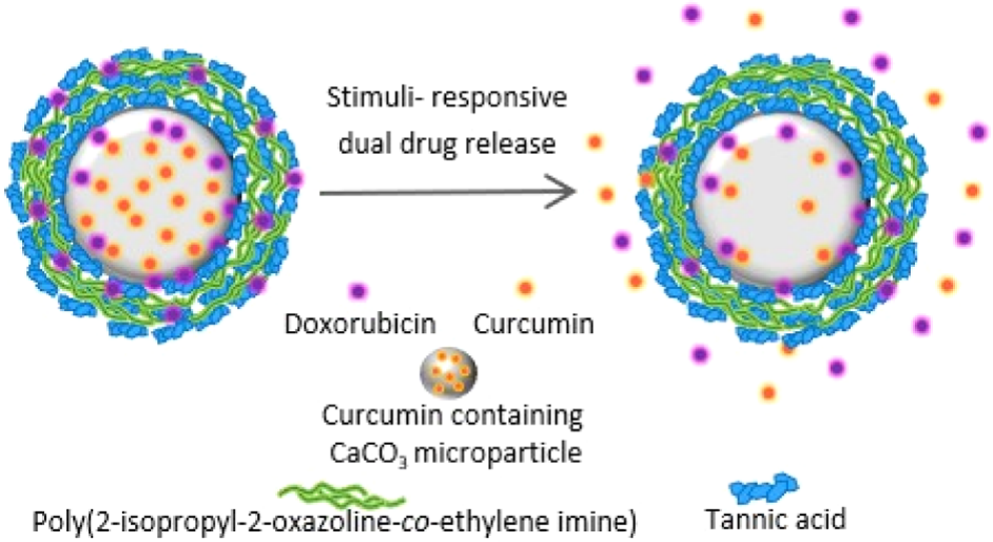

This study reports, first, on the preparation and cross-linking of multilayers composed of poly(2-isopropyl-2-oxazoline-*co*-ethyleneimine) (PiPOX-PEI) and tannic acid (TA). PiPOX was synthesized by cationic ring-opening polymerization (CROP) and partially hydrolyzed, yielding a random copolymer PiPOX-PEI. It was then coassembled at the surface with TA using the layer-by-layer (LbL) technique. Multilayers were exposed to NaIO_4_ solution to induce covalent bond formation between PEI units of PiPOX-PEI and TA. Cross-linking with NaIO_4_ enhanced the stability of the multilayers, especially under basic conditions. Second, the potential of PiPOX-PEI and TA multilayers as a stimuli-responsive dual drug-releasing platform was examined using curcumin (CUR) and doxorubicin (DOX) as model drugs. These drugs were chosen as they can act in a combinatorial manner to increase cell death. The surface of CUR-containing CaCO_3_ microparticles was modified with PiPOX-PEI and TA multilayers and postloaded with DOX. We found that LbL particles could release DOX in a pH-responsive manner, whereas temperature-induced release was observed only when the temperature was raised above 40 °C. The DOX and CUR released from the LbL particles could act synergistically on HCT-116 cells. Cross-linking increased the DOX release from LbL particles but decreased the CUR release from the core. Corroborating the release data, the synergy observed with the non-cross-linked particles was lost with the cross-linked particles, and the decrease in the viability of HCT-116 cells was attributed mainly to the release of DOX. Overall, we describe here NaIO_4_-induced cross-linking of PiPOX-PEI/TA LbL films, the effects of pH, temperature, and cross-linking on DOX and CUR release from multilayers, and comparison of the combinatorial effect of DOX and CUR for cross-linked and non-cross-linked LbL microparticles through cell viability assays.

## 1 Introduction

Poly(2-alkyl-2-oxazoline)s (PAOXs) are polyamides, which are synthesized through cationic ring-opening polymerization of 2-oxazolines.^1^ PAOXs were discovered in the 1960s,^2^ and since then, have been extensively studied due to their unique properties arising from their polyamide backbone and structural versatility. PAOXs draw attention to biomedical applications due to their biocompatible, antifouling, stealth, and nontoxic properties.^3^ Besides, they can be chemically modified through their side groups for the preparation of antimicrobial surfaces, micelle drug carriers, and drug–polymer conjugates.^2^ Among PAOXs, poly(2-isopropyl-2-oxazoline) exhibits lower critical solution temperature (LCST) behavior close to body temperature (36–39 °C^4^), thus it has become of interest for biomedical fields including chemosensor,^5^ drug delivery^6^, and tissue engineering^7^ applications. Of note, PiPOX is the constitutional isomer of poly(*N*-isopropyl acrylamide) PNIPAM, which is a widely used temperature-responsive polymer (LCST ∼ 32 °C) in biomedical applications. The hysteresis observed for PiPOX during reversible phase change between 26 and 46 °C was reported to be lower than that for PNIPAM, making PiPOX advantageous over PNIPAM for the preparation of reversible/switchable smart materials.^8^

Layer-by-layer (LbL) self-assembly is a thin film fabrication technique based on alternating deposition of interacting polymers onto a surface. It is preferred especially for biomedical applications due to the possibility of using water-soluble polymers to functionalize surfaces or prepare multilayer platforms for the incorporation/release of biologically functional molecules. The lower toxicity of neutral polymers compared to polycations has made hydrogen bonding-driven LbL films more advantageous over electrostatic multilayers.^9^ LbL technology can also be applied to micrometer- and submicrometer-sized colloidal particles. Melamine formaldehyde, silica, polystyrene, and calcium carbonate particles are the most widely used three-dimensional (3D) substrates for LbL deposition.^10^ Among these substrates, CaCO_3_ microparticles are preferred for drug delivery applications due to relatively easy and low-cost synthesis, pH-responsiveness, and properties such as biodegradability, nontoxicity, biocompatibility, and high loading capacity (LC).^11^ Apart from these, calcium carbonate substrates are also advantageous for obtaining LbL hollow capsules due to easy removal through treatment with ethylenediaminetetraacetic acid (EDTA) or exposure to slightly acidic conditions.

The increasing interest toward PAOXs and the numerous advantages of the LbL technique has drawn attention toward the preparation of PAOX-based multilayer assemblies. After the first studies on LbL deposition of various PAOXs with polyacids,^12−15^ the potential applications of PAOX-based multilayers such as the preparation of antiadhesive surfaces,^16−18^ stimuli-responsive drug/macromolecule releasing platforms,^6,17^ antibacterial surfaces,^19^ intracellularly degradable^20^ polymer capsules have been reported. Although there are several studies concerning the functionalization of surfaces using PAOXs through the LbL self-assembly technique, there are still challenges to address in the use of PAOX-based LbL films for biomedical applications. For example, PAOXs are neutral polymers, and their LbL films are constructed through hydrogen bonding interactions. This may lead to long-term stability problems in biomedical applications since hydrogen bonding is sensitive to pH, ionic strength, and temperature of the environment. Stabilization of multilayers composed of PAOXs and poly(carboxylic acid)s has been reported. Partially hydrolyzed poly(2-ethyl-2-oxazoline) (PEOX-PEI) and poly(acrylic acid) multilayers were stabilized by thermal cross-linking through amide bond formation.^16,21^ Stabilization of multilayers composed of poly(methacrylic acid) and PAOXs containing alkene and alkyne moieties has been achieved through thiol–ene and copper-catalyzed azide–alkyne cycloaddition reactions, respectively.^18^ Cross-linking of thiol-containing PEOX and poly(methacrylic acid) through disulfide formation was also reported.^20^

Different from the studies mentioned above, we report here the cross-linking of hydrogen-bonded PAOX-based multilayers, where the hydrogen-donating polymer is a polyphenol. Specifically, PiPOX was partially hydrolyzed, yielding a random copolymer PiPOX-PEI which was coassembled at the surface with tannic acid (TA). Multilayers were exposed to NaIO_4_ solution to induce covalent bond formation between the PEI units of PiPOX-PEI and TA. We next examined whether this system can be used as a platform for the delivery of multiple drugs. Doxorubicin (DOX) is a chemotherapy drug that slows the growth of cancer cells and has been used to treat breast cancer, bladder cancer, lymphoma, and lymphocytic leukemia.^22^ Curcumin (CUR) is a natural polyphenol with anti-inflammatory, antimicrobial, and antioxidant properties.^23^ CUR also exhibits antitumor activity by affecting various biological pathways involved in apoptosis, cell-cycle regulation, tumorigenesis, and metastasis.^24^ When CUR and DOX are used together, the DOX-induced toxicity on the heart, kidney, liver, brain, and reproductive organs was found to diminish due to the antioxidant and apoptosis induction effects of CUR.^25^ Besides, codelivery of DOX and CUR through various delivery platforms has been reported to achieve synergistically enhanced inhibition of cancer cell growth.^26−29^ The number of studies reporting the codelivery of DOX and CUR from LbL microparticles or LbL hollow capsules is limited. A study by Patil and co-workers has shown that chitosan/nanocrystalline cellulose multilayers with DOX postloading could release DOX, whereas the same multilayers could be used to release CUR if nanocrystalline cellulose was loaded with CUR prior to LbL self-assembly.^30^ In this study, we LbL-modified the surface of CUR-containing CaCO_3_ microparticles with PiPOX-PEI and TA and postloaded with DOX. The effects of pH, temperature, and cross-linking on DOX and CUR release from LbL microparticles were examined. Finally, the effect of the combinatorial release of CUR and DOX on the viability of HCT-116 cells was compared with particles containing DOX or CUR alone. Moreover, comparison of the combinatorial effect of DOX and CUR for cross-linked and non-cross-linked LbL microparticles was also discussed. The fundamental information generated in this study may form a basis for the development of stimuli-responsive carriers for drug release.

## 2 Materials and Methods

### 2.1 Materials

Phosphate-buffered saline (PBS) (tablet), sodium carbonate (Na_2_CO_3_) (powder, ≥99.5%, ACS reagent), CUR, poly(sodium 4-styrenesulfonate) (PSS) (70,000 g/mol), poly(vinylpyrrolidone) (PVP) (10,000 g/mol), ethanolamine (>99%), cadmium acetate dihydrate (98%), α-bromoisobutyryl bromide (98%), acetonitrile (>99.9%), 2-butanol (>99%), branched poly(ethyleneimine) (BPEI) (25,000 g/mol), and dimethyl sulfoxide (DMSO) were purchased from Sigma-Aldrich Chemical Co. DOX hydrochloride was purchased from European Pharmacopoeia RSs. Calcium chloride anhydrous (CaCl_2_) was purchased from CARLO ERBA Reagents. Sodium dodecyl sulfate (SDS) was purchased from BioShop Canada Inc. Ethanol (≥99.9%) was purchased from Isolab Chemicals. Hydrochloric acid (HCl) fuming (37%), sulfuric acid (H_2_SO_4_) (98%), sodium dihydrogen phosphate dihydrate (NaH_2_PO_4_·2H_2_O), TA (1701 g/mol), sodium hydroxide (NaOH) (pellet), and Spectra/Por 7 regenerated cellulose dialysis membrane (molecular weight cut off: 3.5 kDa) were purchased from Merck Chemicals. Deionized (DI) H_2_O was purified by passage through a Milli-Q system (Millipore) at 18.2 MΩ. Roswell Park Memorial Institute (RPMI)-1640 medium, fetal bovine serum (FBS), and l-glutamine were purchased from Biological Industries (Beit Haemek, Israel). HEK293T cells were a kind gift from Assist. Prof. Ahmet Acar (METU). HCT-116 cells were purchased from DKFZ (Heidelberg, Germany). Plasmocin was purchased from InvivoGen (San Diego, CA). 3-(4,5-Dimethylthiazol-2-yl)-2,5-diphenyltetrazolium bromide Vybrant MTT assay kit was purchased from Invitrogen (Carlsbad, CA). Coomassie protein assay reagent and M-PER mammalian protein extraction reagent were purchased from Thermo Scientific. Complete mini-ethylenediaminetetraacetic acid (EDTA)-free protease inhibitor cocktail and PhosSTOP phosphatase inhibitor were purchased from Roche, Germany. Dulbecco’s phosphate-buffered saline (D-PBS) was purchased from Biowest (Nuaillé, France). T25, T75, and other cell culture plates were purchased from Greiner Bio-One (Kremsmünster, Austria).

#### 2.1.1 Synthesis of 2-Isopropyl-2-oxazoline (iPOX)

The same procedure with our recent study was used.^31^ Ethanolamine (0.052 mol, 3.52 mL) was mixed with isobutyronitrile (0.043 mol, 3.9 mL). Cadmium acetate dihydrate (1.08 mmol, 0.29 g) was added to this mixture, followed by refluxing at 130 °C for 20 h under an argon atmosphere. The product was distilled under low pressure at 50 °C and separated from unreacted ethanolamine and isobutyronitrile. It was dried with CaH_2_ and redistilled under vacuum. ^1^H NMR (CDCl_3_, 400 MHz): δ (ppm) = 4.25 (t, 2H, −OCH_2_), 3.75 (t, 2H, =NCH_2_), 2.55 (m, 1H, −CCH), 1.17 and 1.19 (d, 6H, −CH_3_) (Figure S1).

#### 2.1.2 Synthesis of PiPOX

The same procedure with our recent study was used.^31^ iPOX and acetonitrile were dried using CaH_2_. 41 mmol of iPOX was dissolved in 5.35 mL of acetonitrile under an inert atmosphere. 0.4 mmol of α-bromoisobutyryl bromide was added to this solution under stirring. The reaction vessel was placed in an oil bath at 80 °C. After 72 h, the mixture was cooled to room temperature. Polymerization was terminated by adding 1.2 mmol of 2-butanol and the solution was stirred at 80 °C for 2 days. Afterward, the solvent was removed using a rotary evaporator. The product was dissolved in DI water and dialyzed against DI water for 2 days. Finally, the solution was freeze-dried. ^1^H NMR (CDCl_3_, 400 MHz): δ 3.50 (s, 4H, −(CH_2_–CH_2_–N)−), 2.9 and 2.7 (m, 1H, −CH), 1.12 (d, 6H, −CH_3_) (Figure S2). Gel permeation chromatography (GPC) traces of PiPOX: *M*_n_ = 6125 g/mol, PDI 1.2 (Figure S3).

#### 2.1.3 Synthesis of PiPOX-PEI

PiPOX-PEI was synthesized using a procedure described before.^32^ 0.16 g of PiPOX was dissolved in 3 mL of concentrated HCl solution. This solution was stirred at 500 rpm at 100 °C for 6 h. Afterward, the solution was cooled to room temperature.15 mL of 6 M NaOH was added to the polymer solution until it became basic. The polymer solution was dialyzed against DI water for 1 day and freeze-dried. The peak at 2.65 ppm was correlated with the protons associated with the PEI units and indicated successful hydrolysis of PiPOX (Figure S4). The percent hydrolysis of PiPOX-PEI was calculated by the following equation. The degree of hydrolysis was calculated as ∼20%.



#### 2.1.4 Synthesis of CUR-Containing CaCO_3_ Microparticles

Synthesis of CUR-containing CaCO_3_ the microparticles were optimized in our recent study^31^ using a procedure described before with slight modifications.^33^ 25 mg of CUR was dissolved in 5 mL of ethanol. 50 mg of SDS and 50 mg of PVP were added into this solution and mixed on a magnetic stirrer at 1000 rpm for 1 h. Ten milligrams of PSS was added to 5 mL of 0.3 M Na_2_CO_3_ solution and stirred for 1 h at 1000 rpm. SDS/PVP/CUR solution in ethanol was added to 5 mL of 0.3 M Na_2_CO_3_ solution containing PSS and mixed at 1000 rpm for 5 min. Five milliliters of 0.3 M CaCl_2_ solution was added to this mixture in a controlled manner for 40 s (1 mL added after every 8 s) and then mixed for 2 min. CUR-containing CaCO_3_ microparticles were separated by vacuum filtration, washed with 50 mL of DI water, and dried in an oven at 60 °C for 1 h. Figure S5 shows the attenuated total reflectance-Fourier transform infrared (ATR-FTIR) spectrum, X-ray diffraction (XRD) pattern, scanning electron microscopy (SEM) image, and particle size distribution of CUR-containing CaCO_3_ microparticles.

The amount of CUR that was incorporated into CaCO_3_ microparticles was determined by calculating the amount of CUR that remained in the filtrate after the filtration of CaCO_3_ microparticles and subtracting this amount from the amount of CUR added during the synthesis (25 mg). The fluorescence intensity of CUR in the filtrate was too high and lied outside the linear range in the calibration curve. Thus, 25 μL of 15 mL filtrate was diluted at a ratio of ∼ 1:13,000 using ethanol/PBS mixture containing 60% of ethanol by volume. The calibration curve for quantification of CUR amount in CUR-containing CaCO_3_ microparticles is represented in Figure S6. The CUR encapsulation efficiency (EE) % and loading capacity (LC) % of the microparticles were calculated as 29.9 ± 0.8 and 5.5 ± 0.1%, respectively, using the following equations
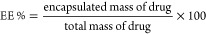

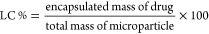


For the control experiments, CaCO_3_ microparticles prepared in the absence of CUR were also used. Briefly, 20 mg of PSS was added to 10 mL of 0.3 M Na_2_CO_3_ solution and mixed at 1000 rpm for 1 h. 0.3 M CaCl_2_ solution was prepared in a separate beaker. Ten milliliter of 0.3 M CaCl_2_ solution was added to 10 mL of 0.3 M Na_2_CO_3_ solution containing PSS and mixed at 1000 rpm for 30 s. CaCO_3_ microparticles were separated by vacuum filtration, washed with 50 mL of DI water, and dried in an oven at 60 °C for 1 h.^34^

### 2.2 Deposition of PiPOX-PEI/TA Multilayers

Prior to film deposition, silicon wafers were immersed in acetone at 50 °C for 10 min. Then, the wafers were immersed in methanol at 25 °C for 3 min, followed by rinsing with DI water and drying under a nitrogen gas flow. The wafers were immersed in concentrated sulfuric acid for 85 min and then rinsed with DI water. Then, the surfaces were immersed in 0.25 M NaOH solution for 10 min and rinsed with DI water. A layer of BPEI was deposited at the surface of the silicon wafers to improve the adhesion of subsequent layers. The wafers were dipped into 0.5 mg/mL BPEI solution (pH 5.5, prepared in 10 mM phosphate buffer solution) for 30 min and then rinsed for 2 min in 10 mM phosphate buffer solution (pH 5.5). 0.2 mg/mL TA solution (pH 6.5) and 0.2 mg/mL PiPOX-PEI (pH 6) solution were prepared by dissolving TA and PiPOX-PEI in 10 mM phosphate buffer. BPEI-coated silicon wafers were first immersed in TA solution for 15 min. Afterward, the silicon wafers were washed twice (2 min each) by immersing the substrates in 10 mM phosphate buffer solution (pH 6.5). Then, the substrates were immersed in PiPOX-PEI solution for 15 min, followed by two rinsing steps using 10 mM phosphate buffer at pH 6 (2 min each) by immersing the substrates in 10 mM phosphate buffer solution (pH 6). This cycle was continued until the desired number of layers was deposited at the surface. Film thickness was measured after each layer by using a spectroscopic ellipsometer.

For cross-linking of PiPOX-PEI/TA multilayers, 14-layer PiPOX-PEI/TA films were immersed into 10 mM NaIO_4_ solution (prepared in 10 mM phosphate buffer) at pH 5 for 5 min and then rinsed twice for 2 min using 10 mM phosphate buffer solution (pH 5).

For QCM-D measurements, a QCM-D sensor with a quartz crystal surface coated with gold (Au) was cleaned via immersion into a mixture composed of DI water/25% ammonia/30% hydrogen peroxide solution with a volume ratio of 5:1:1 at 75 °C for 5 min. The sensor was rinsed with DI water and dried with N_2_ gas. PiPOX-PEI and TA solutions were prepared as described above. The sensor was pre-equilibrated with pH 6 phosphate buffer (rinsing solution of PiPOX-PEI) for a minimum of 30 min to establish a stable baseline. PiPOX-PEI, TA, and rinsing solutions were purged into the sensor chamber at a flow rate of 150 μL/min. Throughout the measurements, the temperature was set to 22 °C. For cross-linking, 10 mM NaIO_4_ (pH 5 phosphate buffer) was purged into the sensor for 5 min and rinsed for 5 min with pH 5 phosphate buffer after deposition of the multilayers.

For UV–vis spectroscopy measurements, multilayers were deposited onto a quartz substrate, and the absorbance of multilayers was measured.

### 2.3 Stability of Multilayers

Cross-linked and non-cross-linked 14-layer PiPOX-PEI/TA films were immersed into PBS at either increasing or decreasing pH. The duration of immersion was 30 min for each pH. The temperature of the solutions was 25 °C. Multilayers were rinsed for 2 min using 10 mM phosphate buffer prior to ellipsometric thickness measurements. For time-dependent evolution of thickness measurements, 14-layer PiPOX-PEI/TA films were immersed in PBS at pH 7.4/37 °C and pH 5.5/37 °C. The films were taken out from the solutions at specific time intervals and rinsed using 10 mM phosphate buffer for 2 min prior to thickness measurements. Fractions retained at the surface were calculated by dividing the thickness of the multilayers by the initial thickness.

### 2.4 Deposition of PiPOX-PEI/TA Multilayers onto CUR-Containing CaCO_3_ Microparticles

Ten milligrams of CaCO_3_ microparticles were placed in an Eppendorf tube and dispersed in 2 mL of 10 mM phosphate buffer solution (pH 6.5) for 1 h using a vortex shaker. Microparticles were precipitated through centrifugation at 3500 rpm for 1 min. The precipitate was dispersed for 20 min in 2 mL of 1 mg/mL TA solution (prepared in 10 mM phosphate buffer solution at pH 6.5) using a vortex shaker. Microparticles were precipitated by centrifugation at 3500 rpm for 1 min. For rinsing, the precipitate was dispersed in 10 mM phosphate buffer solution (pH 6.5) for 1 min using a vortex shaker and precipitated through centrifugation at 3500 rpm for 1 min. The rinsing process was repeated twice. For the second layer, the precipitated microparticles were dispersed in 2 mL of 0.5 mg/mL PiPOX-PEI solution (prepared in 10 mM phosphate buffer solution at pH 6) for 15 min using a vortex shaker. Microparticles were precipitated through centrifugation at 3500 rpm for 1 min. For rinsing, the precipitate was dispersed in 10 mM phosphate buffer solution (pH 6) for 1 min using a vortex shaker and precipitated through centrifugation at 3500 rpm for 1 min. The rinsing process was repeated two times. This cycle continued until five layers of PiPOX-PEI/TA were deposited onto CaCO_3_ microparticles. The deposition time was 15 min for all layers except the first TA layer which was 20 min. 0.5 mg/mL TA solution was used for the second and third TA layers.

For cross-linking, LbL-coated microparticles were dispersed in 10 mM NaIO_4_ solution (prepared in 10 mM phosphate buffer at pH 5) for 5 min using a vortex shaker. The particles were precipitated through centrifugation at 3500 rpm for 1 min and then rinsed twice (2 min each) using 10 mM phosphate buffer solution at pH 5.

### 2.5 Postloading and Release of DOX into/from LbL-Coated Microparticles

Five mg of CUR-containing CaCO_3_ microparticles coated with five layers of PiPOX-PEI/TA were dispersed in 1 mL of 0.1 mg/mL DOX solution (prepared in 10 mM phosphate buffer at pH 7.4) and vortexed at 1600 rpm for 1 h. Of note, cross-linking was performed prior to DOX loading in the case of cross-linked LbL particles. The particles were precipitated by centrifugation at 3500 rpm for 1 min and redispersed in 10 mM phosphate buffer at pH 7.4 to remove weakly bound DOX molecules. The supernatant was collected, and the amount of DOX was determined by using calibration curves. The amount of DOX loaded into LbL-coated CUR-containing CaCO_3_ microparticles was approximated by subtracting the amount of DOX in the supernatant from the amount of DOX in the postloading solution. The calibration curve for quantification of the loaded DOX amount is represented in Figure S7. The DOX EE % and LC % of the non-cross-linked and cross-linked particles were calculated using the equations described in Section 2.1.4 and presented in Table 1.

**Table 1:** DOX EE % and LC % of the Non-Cross-Linked and Cross-Linked Particles

sample	EE %	LC %
DOX loading to non-cross-linked LbL-coated particles	98.1 ± 0.1	2.8 ± 0.3
DOX loading to cross-linked LbL-coated particles	97.7 ± 2.9	3.9 ± 0.1

For DOX release, 3 mg of microparticles were dispersed in 1.5 mL of PBS and mixed at 300 rpm on a magnetic stirrer. DOX release from non-cross-linked particles was followed at pH 5.5/25 °C; 7.4/25 °C; 5.5/37 °C; 7.4/37 °C and 5.5/42 °C. DOX from cross-linked particles was followed at pH 5.5/37 °C. The microparticles were precipitated at specific time intervals. The supernatant was separated and diluted at a ratio of 1:10 using PBS prior to fluorescence intensity measurements (λ_excitation_ = 490 nm and λ_emission_ = 592 nm, slit widths were 10 nm) because of the high concentration of DOX which did not lie at the linear region in the calibration curves. LbL microparticles were redispersed in 1.5 mL of fresh PBS solution. The cumulative amount of DOX released from LbL microparticles was calculated by summing up instant and all loss amounts.

### 2.6 CUR Release from LbL-Coated Microparticles

CUR release was followed from PiPOX-PEI/TA-coated CUR-containing CaCO_3_ microparticles. Three milligram LbL-coated CUR-containing CaCO_3_ microparticles were dispersed in 1.5 mL of ethanol/PBS mixture for 5 min using a vortex shaker. Release of CUR was followed in ethanol/PBS mixture (20% ethanol by volume) at either pH 7.4/37 °C or 5.5/37 °C. Particles were stirred at 300 rpm using a magnetic stirrer. The particles were precipitated at specific time intervals. 0.3 mL of the supernatant was diluted to 1 mL using ethanol/PBS mixture (20% ethanol by volume). Then, this solution was rediluted to 2 mL using pure ethanol. For release after 5 h, 1 mL of the supernatant was diluted to 2 mL using pure ethanol. All samples contained 60% ethanol by volume prior to fluorescence intensity measurements at 535 nm (λ_excitation_ = 425 nm and slit width was 10 nm). Of note, LbL microparticles were dispersed in 1.5 mL of fresh ethanol/PBS mixture (20% ethanol by volume) every hour to minimize the effect of degradation of CUR on the quantification. CUR release was quantified using calibration curves. The cumulative amount of released CUR was calculated by summing up instant and all loss amounts. CUR release from cross-linked particles was followed at pH 5.5/37 °C using the same procedure.

### 2.7 Cell Culture and Cell Viability Assays

HCT-116 cells were cultured in RPMI 1640 medium supplemented with 10% FBS, 1 mM sodium pyruvate, 2 mM l-glutamine, and 100 units/mL penicillin–100 g/mL streptomycin in a 37 °C incubator with 95% air and 5% CO_2_. HEK293T cells were grown in DMEM (high glucose: 4.5 g/L) medium supplemented with 10% FBS, 1 mM sodium pyruvate, 6 mM l-glutamine, 1% nonessential amino acids, and 100 units/mL penicillin–100 g/mL streptomycin in a 37 °C incubator with 95% air and 5% CO_2_. The cells were routinely tested for mycoplasma contamination using polymerase chain reaction (PCR) and were treated with a maintenance dose of plasmocin (2.5 μg/mL). The 3-(4,5-dimethylthiazol-2-yl)-2,5-diphenyltetrazolium bromide (MTT) assay (Thermo Fisher) was used to measure cell viability according to the manufacturer’s instructions. To test the effect of free DOX, CUR, or their combination on the viability of cancer cells, HCT-116 cells were seeded in complete medium at a density of 10,000 cells per well and allowed to attach overnight. CUR was dissolved in DMSO, and DOX was dissolved in phosphate-buffered saline. HCT-116 cells were treated with 0, 0.2, 1 and 1.5 μM DOX alone or in combination with 20 μM CUR for 24 h. Following the completion of the treatment period, cell culture medium was aspirated, and 100 μL of a 1.2 mM MTT solution was added to each well. After 4 h, 100 μL of 1% SDS–0.01 M HCl solution was added to the MTT-added wells and incubated for an additional 18 h at 37 °C. The absorbance at 570 nm was measured using a Multiskan-GO microplate reader (Thermo Fisher Scientific).

To test the effect of drug-loaded microparticles on the viability of cancerous and healthy cells, HCT-116 and HEK293T cells were seeded in their respective complete medium at a density of 10,000 cells per well and allowed to attach overnight. The microparticles were first dispersed at a concentration of 500 ppm in complete RPMI medium with constant vortexing for 30 min. Next, each microparticle sample was serially diluted to 100, 50 and 25 ppm with complete RPMI medium. The cells were then treated for 24 h with the microparticles [(i) bare CaCO_3_ microparticles (no CUR, no DOX), (ii) LbL-coated CUR-containing CaCO_3_ microparticles, (iii) DOX postloaded, LbL-coated CaCO_3_ microparticles, (iv) DOX postloaded, LbL-coated CUR-containing CaCO_3_ microparticles, (v) NaIO_4_ treated bare CaCO_3_ microparticles (no CUR, no DOX), (vi) cross-linked LbL-coated CUR-containing CaCO_3_ microparticles, (vii) DOX postloaded, cross-linked LbL-coated CaCO_3_ microparticles, (viii) DOX postloaded, cross-linked LbL-coated CUR-containing CaCO_3_ microparticles at varying concentrations (0, 25, 50, and 100 ppm)]. Following the completion of the treatment period, the cell culture medium containing the microparticles was aspirated and the cells were processed for an MTT assay as described previously.

### 2.8 Calculation of Combinatorial Index (CI) and Synergy of DOX and CUR

The dose–effective curves of each non-cross-linked microparticle loaded with DOX or CUR, or a combination of the two, was used to assess synergism, antagonism, or additive effects. The combination index (CI) value was calculated based on dose–effective parameters of each microparticle loaded either with CUR and DOX alone (*m*1, Dm1, *r*1 and *m*2, Dm2, *r*2) or in combination (*m*3, Dm3, *r*3) using Compusyn software^35^ where *m* = slope (signifies the shape of the curve), Dm = IC_50_ (signifies the potency), and *r* = linear correlation coefficient. These parameters were calculated on the basis of the amount of DOX and CUR released from the non-cross-linked particles and uploaded to the Compusyn software for the determination of the CI and dose reduction index (DRI). CI < 1, CI = 1, and CI > 1 indicate synergistic, additive, antagonist effects, and DRI < 1, DRI = 1, and DRI > 1 indicate nonfavorable dose reduction, no dose reduction, and favorable dose reduction, respectively.

### 2.9 Instrumentation

^1^H NMR spectra of iPOX, PiPOX, and PiPOX-PEI were recorded using a Bruker Spectrospin Avance DPX-400 Ultra shield instrument, operating at 400 MHz. Zetasizer Nano-ZS equipment (Malvern Instruments Ltd., U.K.) was used for hydrodynamic size measurements of PiPOX-PEI which were carried out via dynamic light scattering technique using a cumulants analysis based on the autocorrelation data. ATR-FTIR spectra of CUR-containing CaCO_3_ microparticles and non-cross-linked and cross-linked LbL particles were recorded using a Nicolet iS10 ATR-FTIR spectrometer (Thermo Fisher Scientific, Waltham, MA). XRD pattern was obtained using a Rigaku X-ray diffractometer with a miniflex goniometer operated at 30 kV and 15 mA Cu Kα line (α = 1.54 Å) as the X-ray source. ζ potential measurements were conducted using Zetasizer Nano-ZS equipment (Malvern Instruments Ltd., U.K.). ζ potential values were obtained from electrophoretic mobility values using the Smoluchowski approximation. For vortex mixing of microparticles and LbL particles, a VWR Mixer Pulse Vortex 230 V EU was used. For centrifugation of microparticles, a Hettich Universal 320 centrifuge was used. SEM images of microparticles were obtained using a JSM-6400 SEM (JEOL, equipped with NORAN system 6 X-ray Micro Analysis system and semaphore digitizer, Westhorst, NL). For SEM imaging, samples were prepared by diluting 40 μL of LbL particle solution with 1000 μL of DI water at pH 6.5 and dropping 50 μL of this solution onto a silicon wafer, followed by drying in a vacuum desiccator. Thickness measurements of LbL films deposited onto silicon wafers were followed using an Optosense spectroscopic ellipsometer (OPT-S6000). The changes in frequency and dissipation were followed using a Q-Sense QCM-D Instrument (Initiator model) and sensors with a quartz crystal surface coated with gold (Au) obtained from Biolin Scientific, Q-Sense Sensors, QSX 301. Absorbance measurements of LbL films deposited onto quartz slides were followed using UV–vis spectroscopy (Hitachi U-2800A spectrophotometer). Drug release studies were conducted using a PerkinElmer LS55 Fluorescence Spectrometer.

## 3 Results and Discussion

### 3.1 LbL Deposition of PiPOX-PEI and TA

**Figure 1:**
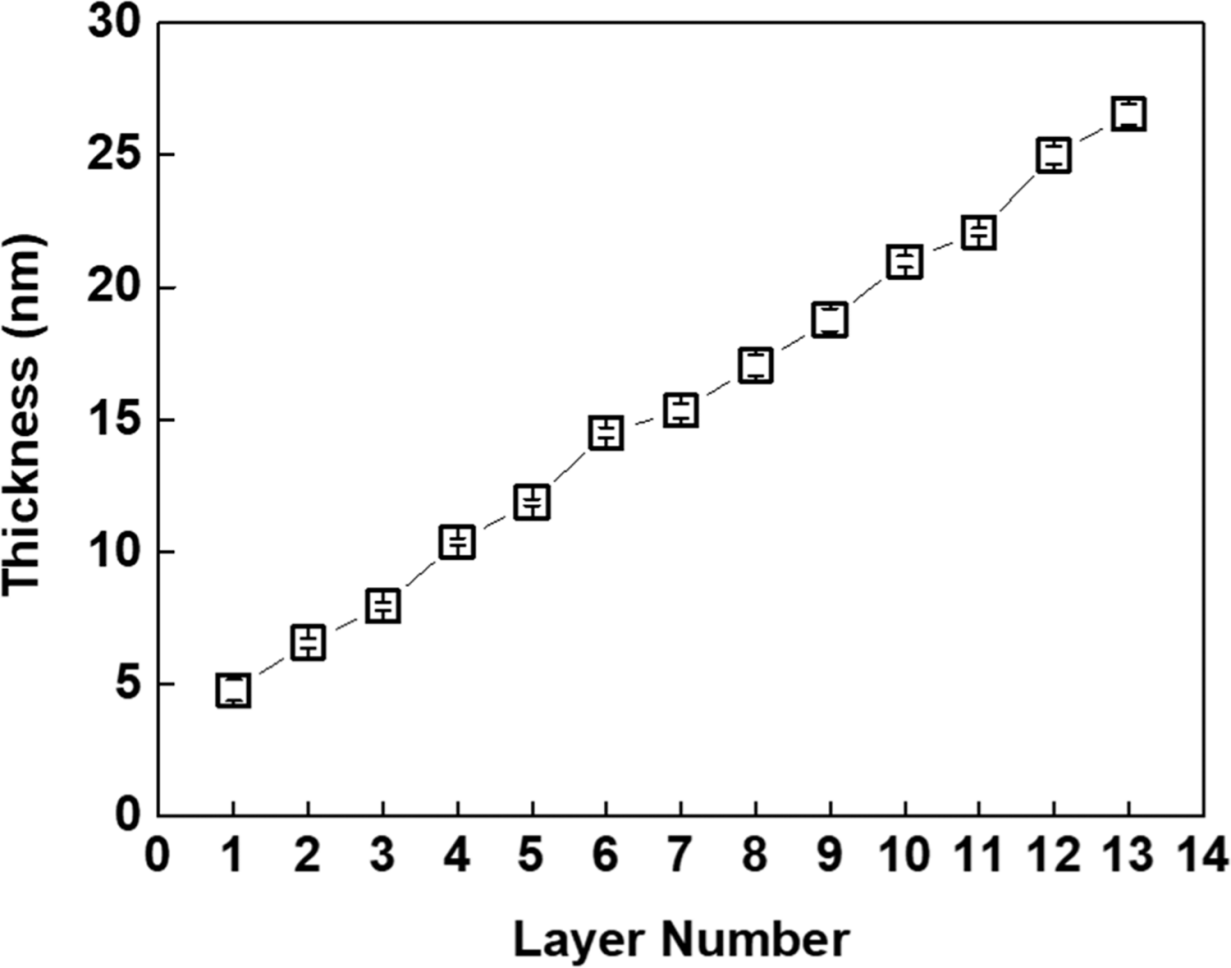
LbL growth of PiPOX-PEI and TA. Multilayers were deposited onto BPEI-coated silicon wafers. The thickness of the BPEI precursor layer was 3 nm and is included in the thickness values.

PiPOX-PEI and TA were deposited at the surface of a silicon wafer at pH 6 and 6.5, respectively. Multilayer growth was followed by measuring the ellipsometric film thickness as a function of layer number (Figure 1). The driving force for multilayer deposition was hydrogen bonding interactions between phenolic hydroxyl groups of TA (for TA p*K*_a,1_ ∼ 6.5 and p*K*_a,2_ ∼ 8^6^) and amide groups of PiPOX-PEI together with electrostatic interactions between the phenolate groups of TA and the protonated secondary amine groups of PiPOX-PEI (p*K*_a_ for secondary amine groups ∼7^36^). The film thickness increased linearly as the layer number increased (∼4 nm increment per bilayer), indicating successful LbL growth of TA and PiPOX-PEI. As discussed in Section 3.3, TA and PiPOX-PEI multilayers were also deposited onto CaCO_3_ microparticles. CaCO_3_ microparticles dissolve below pH 6, thus LbL deposition was performed around pH 6 to ensure the colloidal stability of the microparticles. Of note, although hydrogen bonding interactions between PiPOX and TA weaken at increasing pH, PiPOX and TA multilayers can be constructed at pH 6.^31^ The reason for introducing PEI units to PiPOX was to (i) enhance the association between PiPOX-PEI and TA through electrostatic interactions between PEI and TA at physiologically related conditions; and (ii) provide amine groups for the cross-linking reaction. Of note, LbL deposition of TA and PiPOX under varying pH conditions has been reported by us previously.^6,17,37^

### 3.2 Covalent Cross-Linking of PiPOX-PEI/TA Multilayers

**Figure 2:**
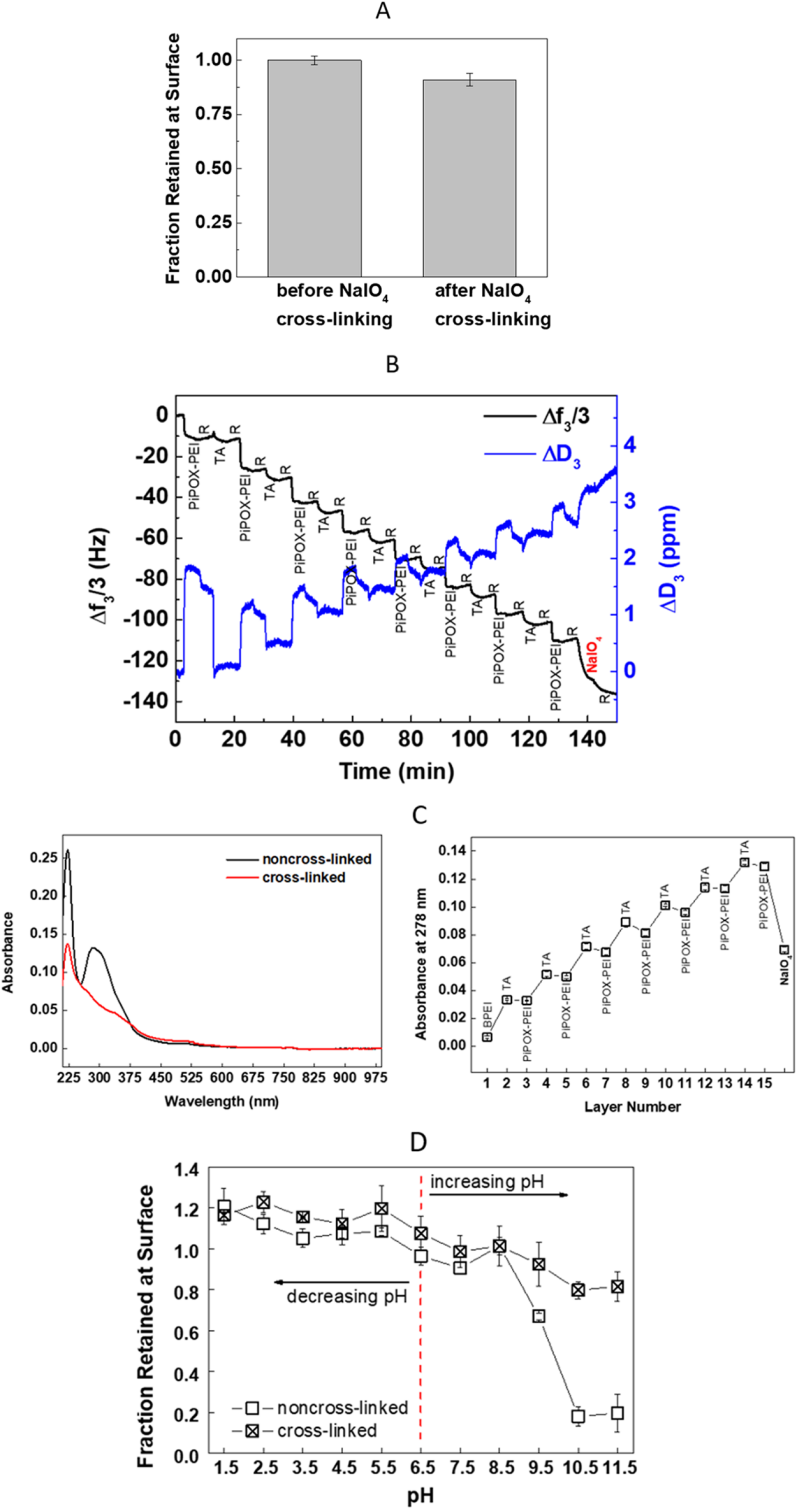
(A) Fraction retained at the surface of the films after NaIO_4_ treatment. (B) Evolution of the change in Δ*f*_3_/3 and Δ*D*_3_ during LbL assembly of PiPOX-PEI and TA and NaIO_4_ treatment at pH 5 and 22 °C. (C) Absorbance spectra of PiPOX-PEI/TA multilayers before and after cross-linking (left panel). The evolution of the absorbance of the peak at 278 nm before and after cross-linking (right panel). (D) The thickness fraction retained at the surface of cross-linked and non-cross-linked multilayers upon exposure to PBS at either decreasing or increasing pH values at 25 °C. The red dotted line represents the starting pH value.

Covalent cross-linking between PiPOX-PEI and TA layers was achieved using NaIO_4_ as the cross-linking agent. 14-Layer PiPOX-PEI/TA films were immersed into a 10 mM NaIO_4_ solution at pH 5.0. After treatment of the films with NaIO_4_, ∼10% loss in film thickness was recorded (Figure 2A). The decrease in thickness was attributed to the loss of polymer layers together with water due to oxidation of TA and partial disruption of hydrogen bonding interactions between TA and PiPOX-PEI. Cross-linking of multilayers was also followed using the QCM-D technique. 14 layers of PiPOX-PEI/TA were deposited *in situ* at the surface of a gold-quartz crystal sensor. Then, 10 mM NaIO_4_ solution (pH 5.0) was purged into the chamber containing PiPOX-PEI/TA-coated sensor for 5 min. As seen in Figure 2B, the frequency decreased upon both deposition of the layers and NaIO_4_ treatment, indicating an increase in the mass-deposited at the surface. The dissipation increases when a soft film attaches to a surface.^38^ Accordingly, the dissipation increased as the PiPOX-PEI/TA multilayers were deposited on the surface. We recorded a further increase in the dissipation upon cross-linking. This might be due to the conversion of phenolic hydroxyl groups to quinones upon treatment with NaIO_4_ solution which might have led to the entrapment of a greater amount of water molecules within the multilayers due to stronger hydrogen bonding between carbonyl groups of quinones and water than that between phenolic hydroxyl groups of TA and water.

Multilayer growth and cross-linking of multilayers were also monitored by using UV–vis spectroscopy (Figure 2C, left). Multilayers were deposited onto the quartz surface. TA exhibits two peaks at 214 and 276 nm in acidic conditions, associated with the neutral form of TA. When the pH of TA solution is increased, two peaks emerge at 245 and 322 nm, associated with the ionized form of TA.^6^ In light of this information, the peaks at 222 and 278 nm in the absorbance spectrum of the non-cross-linked film were associated with the phenolic hydroxyl groups of TA. Nevertheless, the broad absorption peak at 278 nm suggested the presence of another peak around 320 nm, which was correlated with the phenolate groups of TA. The absorbance at 278 nm increased after every TA layer deposition (Figure 2C, right). The sharp decrease in the absorbance at 278 nm upon cross-linking was due to the oxidation of phenols to quinones, resulting in a decrease in the number of phenolic hydroxyl/phenolate groups within the multilayers.

Lastly, the formation of covalent cross-links between the layers was indirectly verified through the difference in the stability of the cross-linked and non-cross-linked films. A 14-layer cross-linked and non-cross-linked PiPOX-PEI and TA films were exposed to PBS at either decreasing or increasing pH at 25 °C. Figure 2D compares the thickness fraction retained at the surface as a function of pH. Fractions were calculated by dividing the thickness by the initial film thickness. Both cross-linked and non-cross-linked films showed an increase in thickness as the pH was decreased. In addition to the salt ions penetrating from the solution into the multilayers, the enhanced hydrophilicity of the polyelectrolytes when paired with the salt ions^39^ might have increased the amount of water entrapped within the film and accounted for the thickness increase. When the non-cross-linked films were exposed to increasing pH conditions, the ionization of TA and loss of hydrogen bonding interactions between PiPOX-PEI and TA were responsible for the onset of dissolution recorded at pH 9.5. Multilayers were totally erased from the surface at pH of 10.5. On the other hand, ∼80% of the cross-linked film was retained at the surface even at pH 11.5. The enhanced stability of the cross-linked film was attributed to the oxidation of phenolic hydroxyl groups of TA to quinone groups by IO_4_^–^ anions and the formation of covalent bonds between the quinone groups and the secondary amine groups of PEI units. There are two possible mechanisms for the cross-linking reaction of the amine groups and quinone groups. One of them is Michael addition, suggesting formation of the N–C bond. The other one is the Schiff Base reaction, suggesting C=N bond formation.^40^ Cross-linking of multilayers containing TA and amine-bearing polycations using NaIO_4_ has been reported earlier.^41,42^

#### 3.2.1 Long-Term Stability of PiPOX-PEI/TA Multilayers

**Figure 3:**
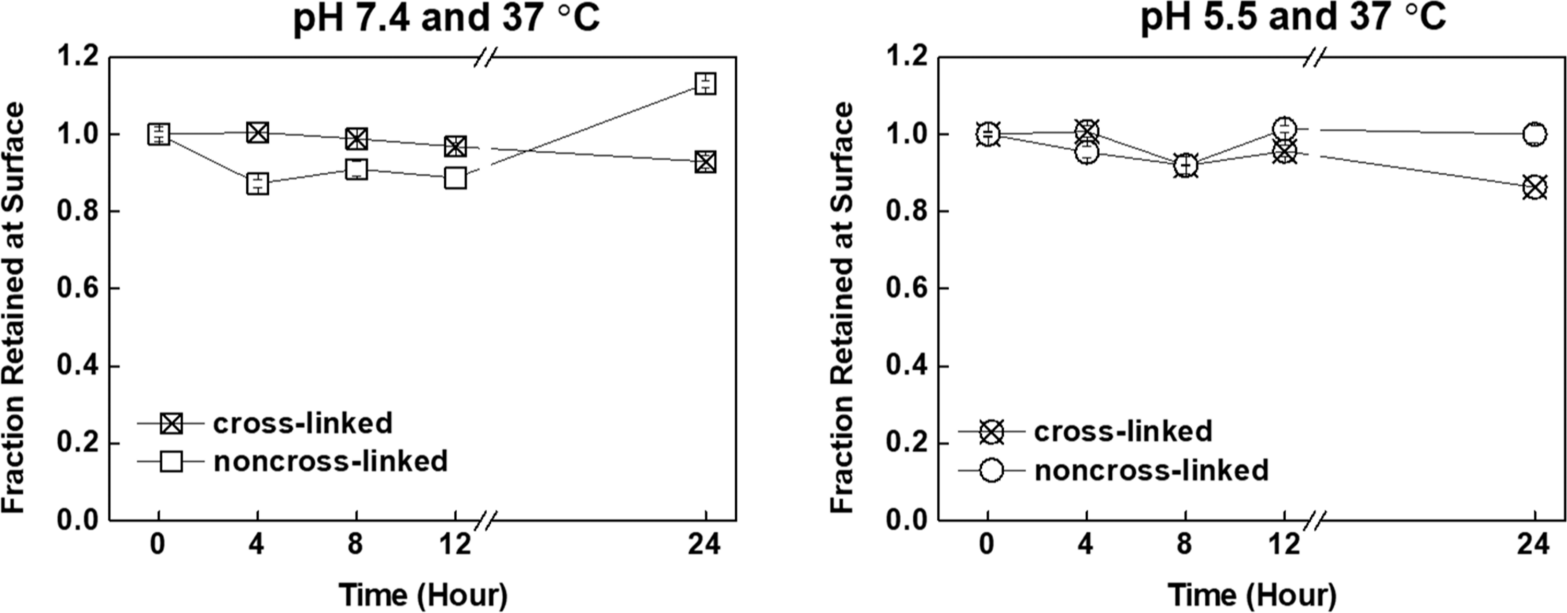
Time-dependent variation of the fraction retained at the surface of non-cross-linked and cross-linked films immersed in PBS at pH 7.4/37 °C and 5.5/37 °C.

The long-term stability of cross-linked and non-cross-linked PiPOX-PEI/TA films was examined under conditions at which drug release studies were conducted. As will be discussed in Section 3.3, two different anticancer drugs were encapsulated in LbL-coated CaCO_3_ microparticles. Considering the acidic nature of tumor tissues, drug release studies were investigated at both pH 7.4 and 5.5 at 37 °C. 14-layer films were prepared and separately immersed into PBS at pH 7.4/37 °C and 5.5/37 °C. PiPOX-PEI/TA films were removed from the solution at specific time intervals, and the thickness of the remaining film was divided by the initial thickness of the multilayers to calculate the fraction retained at the surface (Figure 3). The thickness of the non-cross-linked film decreased (by ∼10%) in the first 4 h at pH 7.4 and 37 °C due to ionization of TA with increasing pH and weakening of the hydrogen bonding interactions among the layers. No further loss was recorded in the measurements conducted after 8 and 12 h. However, the thickness measurement displayed an increase of ∼25% after 24 h. This increase was attributed to partial loss of layers from the surface which resulted in a relatively loose film structure and entrapment of a greater amount of water molecules within the multilayers. The extent of loss and the subsequent increase in thickness after 24 h were smaller at pH 5.5 and 37 °C than at pH 7.4 and 37 °C. The greater stability at pH 5.5 was attributed to the enhanced hydrogen bonding interactions among the layers as TA further protonated with decreasing pH, leading to more strongly associating layers. Importantly, we did not record any significant thickness change for cross-linked films upon exposure to PBS at both pH 7.4 and pH 5.5.

### 3.3 Preparation of DOX- and CUR-Containing LbL Microparticles

In order to decide on the amount of DOX and CUR to load in the LbL microparticles, we first carried out a dose–response effect of free DOX on HCT-116 colon cancer cells to determine the IC_50_ value. Previous studies have reported a wide range of IC_50_ values of DOX for HCT-116 cells, ranging from 0.4 to about 1 μM.^43,44^ Therefore, we used a range of 0–1.5 μM DOX for 24 h. We observed that the cells had about 55% cell viability with 1.5 μM DOX (Figure S8). Previous studies have shown that the IC_50_ value of CUR for HCT-116 cells was between 10 and 25 μM.^45^ Therefore, we directly tested the effect of 20 μM CUR with the different concentrations of DOX (0–1.5 μM). We observed that with 20 μM CUR alone the cells had around 57% survival; the addition of various concentrations of DOX to 20 μM CUR could increase cell death, albeit modestly. Several previous studies have shown that CUR can increase the sensitivity of cancer cells to DOX.^46^ Preliminary studies carried out with the LbL microparticles (100 ppm) showed that the amount of CUR that could be loaded successfully was around 15 μM.

**Figure 4:**
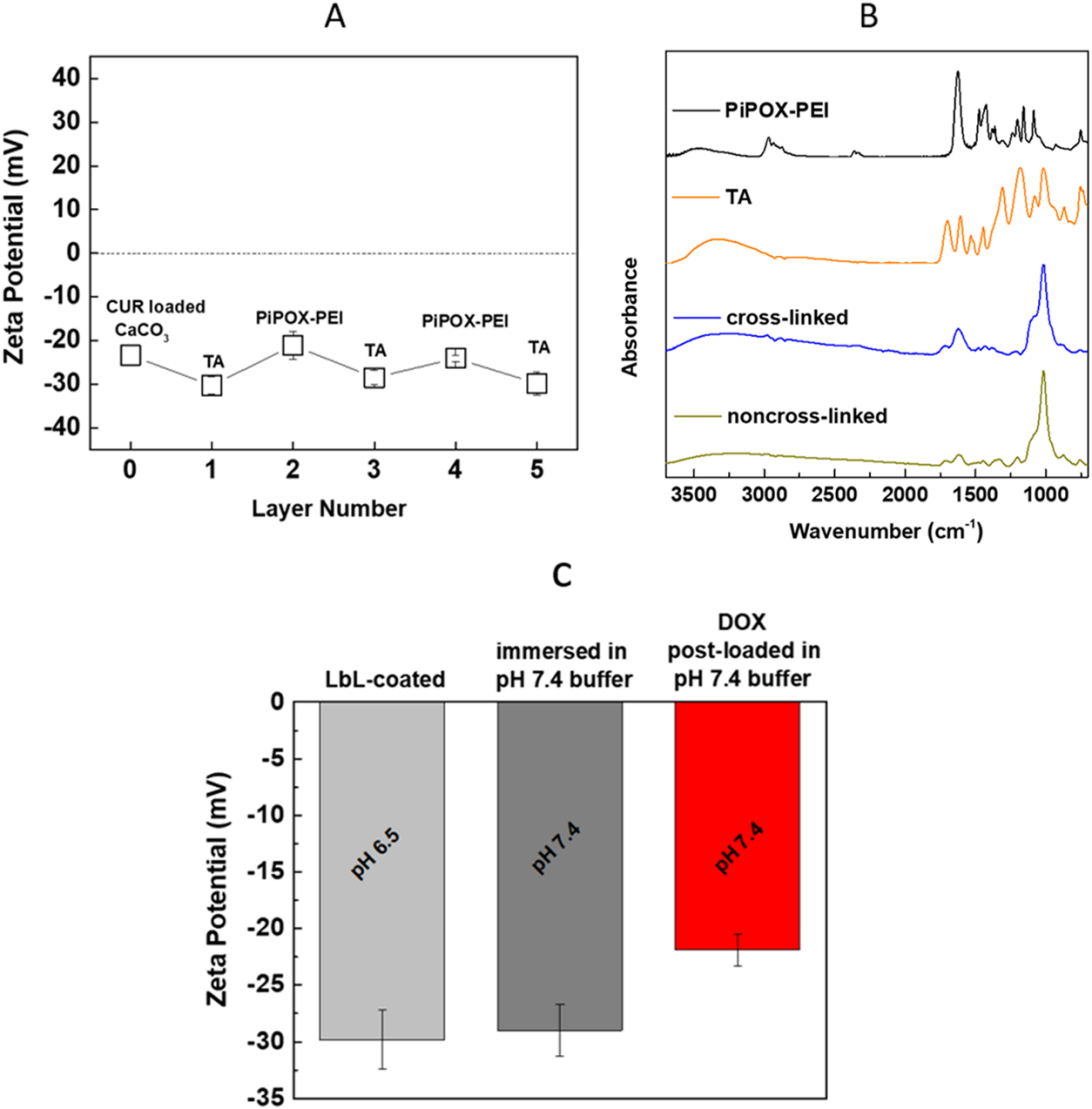
(A) Evolution of ζ potential as a function of layer number. (B) ATR-FTIR spectrum of cross-linked vs non-cross-linked five-layer PiPOX-PEI/TA-coated CUR-containing CaCO_3_ microparticles. TA and PiPOX-PEI spectra were drawn for comparison. (C) ζ potential values of CUR-containing CaCO_3_ microparticles after LbL coating (topmost layer: TA, deposition at pH 6.5), after immersion of LbL-coated particles into phosphate buffer at pH 7.4 and LbL-coated particles after DOX postloading in phosphate buffer at pH 7.4.

TA and PiPOX-PEI layers were deposited onto CUR-containing CaCO_3_ microparticles with sizes ranging between ∼2 and 4 μm. LbL growth was followed by measuring the ζ potential of the microparticles after the deposition of each layer (Figure 4A). The change in ζ potential was assumed as an indication of LbL growth. The mean ζ potential changed from −23.4 ± 2.4 to −30.3 ± 2.0 mV after the first TA layer deposition due to phenolate groups of TA (p*K*_a,1_ ∼ 6.5 and p*K*_a,2_ ∼ 8^6^). The driving force for the deposition of TA onto CaCO_3_ microparticles was electrostatic interactions between Ca^2+^ and phenolate groups of TA and hydrogen bonding interactions between oxygen atoms of carbonate anions and phenolic hydroxyl groups of TA. Of note, CaCO_3_ microparticles were synthesized in the presence of PSS, which is located inside and on the surface of CaCO_3_ microparticles.^47^ Therefore, hydrogen bonds between the SO_3_^2–^ units of PSS and the OH groups of TA together with π–π stacking interactions between the aromatic rings of TA and the phenyl rings of PSS might have contributed to the deposition of TA at the surface. After the deposition of the second layer (PiPOX-PEI), the ζ potential was recorded as −21.1 ± 3.2 mV. The shift of the ζ potential toward a less negative value was due to partial compensation of the negative charge on the microparticles by positively charged PEI units as well as screening of the negative charge by the neutral PiPOX units. The ζ potential oscillated in the negative region during the LbL process with more and less negative values recorded after the deposition of TA and PiPOX-PEI layers, respectively. The size distribution shifted somewhat to higher values after LbL assembly possibly due to enhanced hydrophobic association among the LbL particles upon modification with polymers (Figure S9).

Cross-linking of PiPOX-PEI/TA-coated CUR-containing CaCO_3_ microparticles resulted in the oxidation of CUR. The orange color of the LbL microparticles immediately turned purple when dispersed in 10 mM NaIO_4_ solution. To confirm that the color change was associated with the CUR in the microparticles, we dispersed uncoated CUR-containing microparticles in NaIO_4_ solution of varying concentrations for 5 min. The particles were precipitated and redispersed in an ethanol/PBS mixture (20% ethanol by volume), and the fluorescence intensity of CUR released from the particles was compared. The fluorescence intensity of CUR released from the particles decreased with increasing concentration of NaIO_4_ solution (Figure S10). The decrease in fluorescence intensity can be explained by the oxidation of methoxyphenol groups of CUR to quinone groups, resulting in the loss of aromaticity/conjugation in CUR.^48^ The primary degradation pathway of CUR was reported to be autoxidation at physiological pH^49^ and the biological activity was found to be provided by CUR rather than its degradation products.^50^

ATR-FTIR spectra of the non-cross-linked and cross-linked CUR-containing LbL-coated CaCO_3_ microparticles are presented in Figure 4B. The spectra of TA and PiPOX-PEI are plotted for comparison. The non-cross-linked and cross-linked LbL-coated particles showed a characteristic peak of TA at 1715 cm^–1^ associated with (C=O) stretching vibration.^51^ The peaks between 1000 and 1400 cm^–1^ were related to C–O stretching vibrations of TA and C–C/C–H vibrations of both TA and PiPOX.^37^ The peaks between 2800 and 3000 cm^–1^ were assigned to antisymmetric C–H stretching of –C(CH_3_)_2_, antisymmetric C–H stretching of –CH_2_–, and symmetric C–H stretching of –C(CH_3_)_2_ of PiPOX.^52,53^ The peak around 1202 cm^–1^ was correlated with the absorption of C–N groups of the PiPOX backbone.^52^ The decrease in the intensity of the peak at 1327 cm^–1^ (C–O stretching vibration) after cross-linking was attributed to the oxidation of phenolic hydroxyl groups of TA to the *o*-quinone structure by NaIO_4_.^54^ The increase in the intensity of the peak at 1621 cm^–1^ (C=C mode of the quinoid ring) upon cross-linking might also be attributed to the oxidation of phenols to quinones.^55^ Of note, a new vibrational band was not distinguished in the spectrum after cross-linking, possibly due to the low hydrolysis degree of PiPOX-PEI and the number of cross-linking points.

**Scheme 1:**
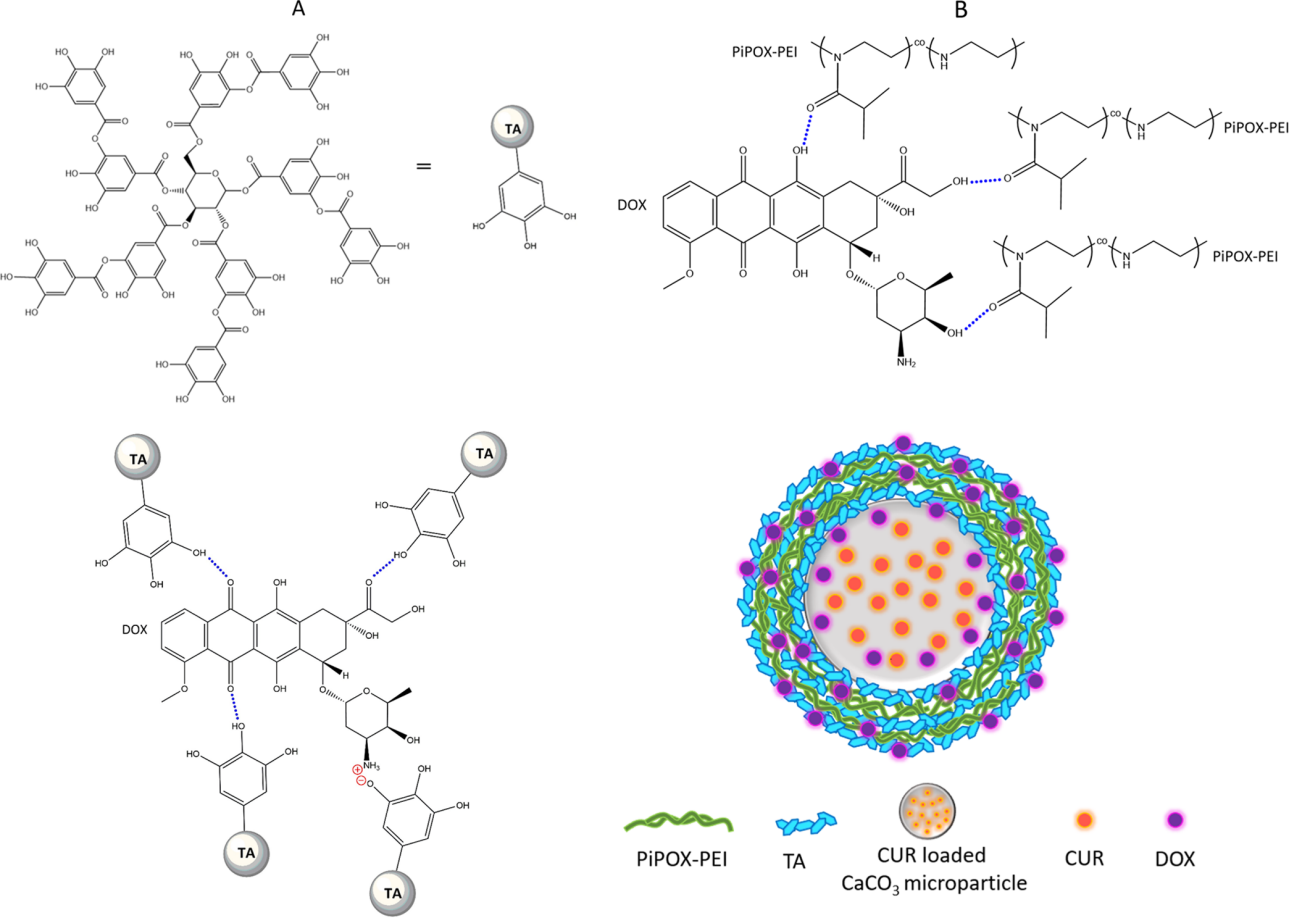
(A) Schematic Representations of Interactions between DOX and TA; (B) DOX and PiPOX-PEI Blue dots represent H-bonding interactions. The pictorial scheme illustrates PiPOX-PEI/TA-coated CUR-containing CaCO_3_ microparticles, postloaded with DOX.

DOX was postloaded into either non-cross-linked or cross-linked LbL microparticles at pH 7.4. The primary driving force for DOX loading into multilayers was electrostatic interactions between protonated amino groups of DOX (p*K*_a_ 8.25^56^) and phenolate groups of TA. In addition, hydrogen bonding interactions between ether oxygens, carbonyl, and hydroxyl groups of DOX and phenolic hydroxyl groups of TA and carbonyl groups of PiPOX-PEI possibly contributed to DOX loading into multilayers. The ζ potential of LbL microparticles shifted to more positive values upon DOX loading due to screening of the negative charge by positively charged DOX molecules (Figure 4C). Scheme 1 presents a schematic representation of the association among DOX/TA and DOX/PiPOX-PEI together with an illustration of CUR and DOX containing LbL microparticles. Of note, in addition to PiPOX-PEI/TA multilayers, DOX might have also been incorporated into the pores of CaCO_3_ microparticles during the postloading process.

### 3.4 *In Vitro* Dual Drug Release from PiPOX-PEI/TA-Coated Microparticles

#### 3.4.1 Release of DOX from PiPOX-PEI/TA-Coated Microparticles

**Figure 5:**
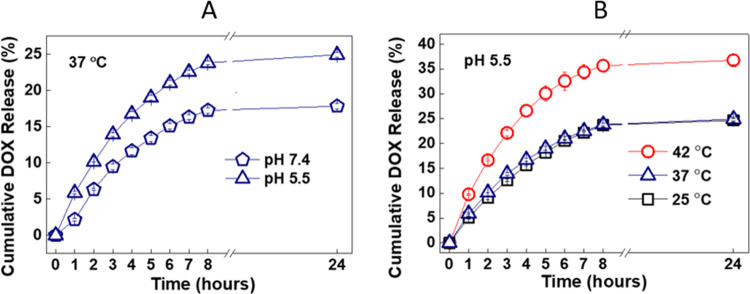
DOX release from PiPOX-PEI/TA-coated CUR-containing CaCO_3_ microparticles into PBS at (A) 37 °C/pH 5.5 and 37 °C/pH 7.4 and (B) 25 °C/pH 5.5, 37 °C/pH 5.5, and 42 °C/pH 5.5.

DOX was followed by measuring the fluorescence intensity of DOX at 592 nm as a function of time. The concentration of DOX released from the particles was calculated using calibration curves prepared under release conditions (Figure S11). The amount of DOX released from LbL-coated microparticles was greater at pH 5.5 than at pH 7.4 at 37 °C (Figure 5A). This can be explained by the protonation of the phenolic hydroxyl groups of TA (p*K*_a,1_ ∼ 6.5 and p*K*_a,2_ ∼ 8) as the pH decreased, which resulted in disruption of electrostatic interactions between TA and DOX, and induced DOX release from multilayers. Of note, CaCO_3_ microparticles partially dissolved as the pH decreased below 6. A ∼10% decrease in weight was recorded when particles were exposed to PBS at pH 5.5. The partial dissolution of CaCO_3_ microparticles might have also contributed to the enhanced release at pH 5.5 by releasing DOX incorporated into the pores of the microparticles.

Nevertheless, the % DOX release was low at pH 5.5, suggesting that a significant amount of DOX remained within the particles. This can be explained by the enhanced ionization of TA in the presence of positively charged DOX molecules, maintaining the electrostatic association between the phenolate groups of TA and protonated amino groups of DOX at pH 5.5. Of note, ionization of polyacids has been reported to enhance in the presence of salt cations in solution^57^ or polycations within the multilayers.^58^ In addition, hydrogen bonding interactions between ether oxygens, carbonyl, and hydroxyl groups of DOX and phenolic hydroxyl groups of TA and carbonyl groups of PiPOX-PEI were possibly responsible for the entrapment of DOX within the multilayers. Apart from the interactions between DOX and polymer layers, DOX which was loaded into CaCO_3_ microparticles might have strongly associated with PSS through both electrostatic (between sulfonate groups of PSS and protonated amino groups of DOX) and hydrogen bonding interactions (between sulfonate groups of PSS and hydroxyl groups of DOX).

DOX release was not affected by increasing the temperature from 25 to 37 °C (see Figure S12 for DOX release at 25 °C). The effect of increasing temperature on DOX release was apparent only when the temperature was raised to 42 °C (Figure 5B). Although, PiPOX exhibits LCST-type phase behavior around 36–40 °C, the lack of temperature-responsive DOX release at 37 °C might be due to an increase in the critical temperature of PiPOX upon hydrolysis. We recently followed the evolution of the hydrodynamic size distribution of PiPOX with respect to increasing temperature. Our observations showed a shift in the size distribution of PiPOX to lower values around the critical temperature which was followed by the emergence of a new peak concerning the formation of large aggregates in the solution at 40 °C due to enhanced hydrophobic interactions between the PiPOX chains.^59^ A similar experiment conducted with PiPOX-PEI showed a similar shift in size distribution to lower values as the temperature was raised from 25 to 45 °C (increased by 2.5 °C) due to the conformational transition of the polymer chains from extended to globular form (see Figure S13). Different from PiPOX, we did not record any new peaks associated with the formation of aggregates which may be considered as an indication of an increase in the critical temperature of PiPOX upon hydrolysis. Apart from the critical temperature, only 2 layers of PiPOX-PEI within the multilayers might not be sufficient to observe a pronounced temperature-responsiveness.

#### 3.4.2 CUR Release from LbL-Coated CUR-Containing Microparticles

CUR release studies were performed in the absence of DOX due to overlapping peaks of DOX and CUR in the emission spectra, which precluded a reliable quantification. Five-layer PiPOX-PEI/TA-coated microparticles were immersed into PBS containing 20% ethanol (by volume) at either pH 7.4/37 °C or pH 5.5/37 °C. Of note, the solubility of CUR is limited in aqueous solution. In addition, their degradation accelerates with increasing pH and temperature, resulting in a decrease in fluorescence intensity. Both of these factors may preclude a reliable quantification of CUR. To increase the solubility of CUR in the release medium for the sake of reliable quantification, a mixture of PBS-ethanol (containing 20% ethanol by volume) was used to determine the amount of CUR released from the particles. Additionally, the release solutions were refreshed every 1 h to minimize the effect of degradation of CUR during quantification. The calibration curve for CUR was also generated in the PBS–ethanol mixture. Although CUR release was confirmed, the amount of CUR may vary, depending on the content of the release medium. Of note, evaluation of the combinatorial effect of DOX and CUR was determined in cell culture experiments using HCT-116 human colorectal cancer cells.

**Figure 6:**
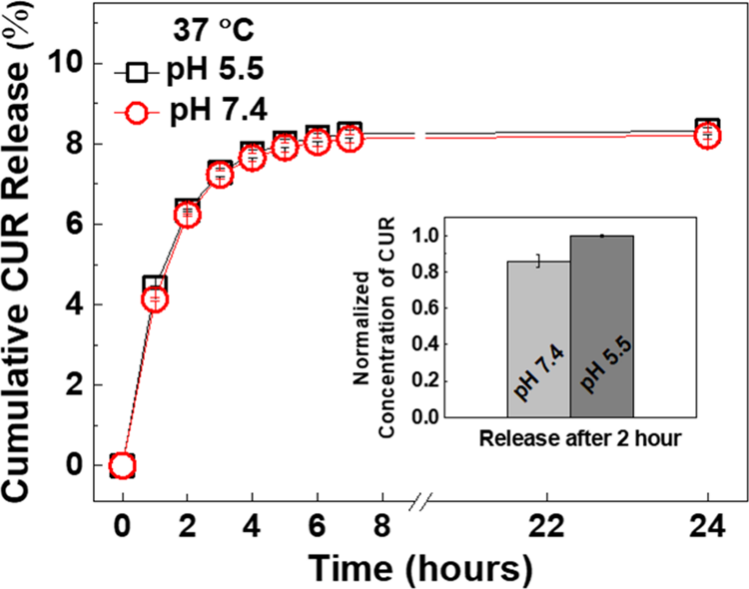
Release of CUR from five-layer PiPOX-PEI/TA-coated CUR-containing CaCO_3_ microparticles at pH 7.4/37 °C and 5.5/37 °C. The inset shows the normalized amount of CUR released from uncoated particles at pH 7.4/37 °C and 5.5/37 °C.

The fluorescence intensity at 535 nm was followed as a function of time and quantified by using calibration curves prepared under release conditions. The calibration curves used for quantification can be found in our recent publication.^31^Figure 6 shows CUR release from LbL-coated microparticles at pH 5.5/37 °C and pH 7.4/37 °C. The majority of CUR was released from the microparticles in the first 3 h. Thereafter, the release slowed down and almost leveled off after 5 h. The amount of CUR released from LbL-coated particles was not affected by the pH of the release medium. This result was different from our recent findings where CUR release from CaCO_3_ microparticles that were embedded into alginate hydrogels was slightly greater at pH 5.5 than at pH 7.4 due to the partial dissolution of CaCO_3_ microparticles at acidic conditions.^31^ The major difference between the two studies with respect to CUR-containing CaCO_3_ microparticles was the LbL coating on the microparticles in this study. To ensure the effect of LbL modification on the lack of pH-responsive CUR release from the particles, we compared the CUR released from uncoated and LbL-coated CUR-containing CaCO_3_ microparticles. Considering the loss of CUR from microparticles during LbL deposition and the variation in the initial amount of CUR in uncoated and LbL-coated particles, the data concerning release from uncoated particles was normalized. To do this, the amount of CUR released at pH 7.4 after 2 h was divided by that at pH 5.5. CUR released from uncoated microparticles was slightly higher at pH 5.5 (see inset in Figure 6). The lack of pH-responsive CUR release with LbL microparticles was attributed to the entrapment of CUR molecules within the multilayers due to enhanced association between hydrophobic CUR and multilayers, which became more hydrophobic as the temperature increased due to LCST-type phase behavior of PiPOX-PEI. It is worth mentioning that the presence of LbL coating may also be effective in preventing the degradation of CUR. The color change observed with uncoated particles after 6 h of exposure to PBS at pH 7.4 and 37 °C was more remarkable than that observed with LbL particles. Further studies need to be conducted to understand the protective effect of the LbL coating on the degradation of CUR.

Of note, % CUR release from LbL particles was low. The following scenarios can be suggested for the low amount of CUR release from LbL particles: (1) CUR-containing CaCO_3_ microparticles was synthesized through the coprecipitation method in this study. This method involves dispersing drug molecules within the solution containing one of the precursor ions (Ca^2+^ or CO_3_^2–^). As Ca^2+^ and CO_3_^2–^ containing solutions are mixed, drug molecules become encapsulated within interior parts or pores of CaCO_3_ particles as precursor ions react to create the precipitates.^60^ However, this at the same time makes CUR release from the particles more challenging. (2) CUR was partially lost from the particles during multilayer deposition and rinsing steps. This was confirmed by the change in the color of the supernatant during the LbL process. Considering the presence of unbound polymers and CUR molecules in the supernatant and their possible association which may affect the fluorescence intensity measurements,^61^ we did not determine the amount of CUR lost from the particles during LbL deposition. The amount of CUR embedded into CaCO_3_ particles was taken as the initial amount during calculations of % CUR release. (3) PiPOX shows LCST-type phase behavior around 36 °C and becomes more hydrophobic near the critical temperature. Increased hydrophobic interactions between CUR and PiPOX might have led to the entrapment of CUR molecules within the layers.

The current study is fundamental in its design. It aimed to contribute to the fundamental understanding of NaIO_4_-induced cross-linking of PiPOX-PEI/TA-coated LbL microparticles and its effect on the release of DOX and CUR from the particles. The combinatorial effect of DOX and CUR that we have observed with the LbL microparticles (Section 3.5), while promising, is among the first reports in the literature on the dual release of these drugs from LbL microparticles. Future studies will be designed to fine-tune the composition of the microparticles to release greater doses of CUR to obtain a stronger combinatorial effect of the two drugs. In addition, new strategies need to be developed to prevent the leakage at physiological pH.

#### 3.4.3 Effect of Cross-linking on DOX and CUR Release from LbL Microparticles

**Figure 7:**
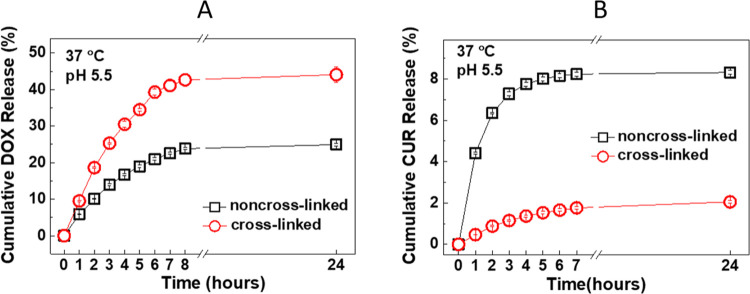
(A) Release of DOX from cross-linked five-layer PiPOX-PEI/TA-coated CUR-containing CaCO_3_ microparticles at pH 5.5/37 °C. Data obtained from non-cross-linked particles was plotted for comparison. (B) Release of CUR from cross-linked five-layer PiPOX-PEI/TA-coated CUR-containing CaCO_3_ microparticles at pH 5.5/37 °C. Data obtained from non-cross-linked particles was plotted for comparison.

DOX release from cross-linked and non-cross-linked five-layer PiPOX-PEI/TA-coated CUR-containing particles was followed in PBS at pH 5.5/37 °C. Cross-linking led to a greater amount of DOX release from the particles (Figure 7A). This was attributed to the more intense structure of cross-linked films which might have prevented the diffusion of DOX to the inner parts of the particles during the postloading process. This possibly resulted in the accumulation of DOX close to the particle surface and provided greater DOX release from the particles.

For CUR release, cross-linked five-layer PiPOX-PEI/TA-coated CUR-containing microparticles were immersed into PBS containing 20% ethanol (by volume) at pH 5.5/37 °C. Release and quantification of CUR were performed as described in Section 2.6. As seen in Figure 7B, in contrast to DOX release, cross-linking reduced the release of CUR from LbL microparticles. The amount of CUR released from non-cross-linked particles was ∼4× times greater than that released from cross-linked particles after 24 h. The lower amount of CUR release from cross-linked particles was attributed to the oxidation of CUR and a decrease in its fluorescence intensity. Besides, cross-linked multilayers with a more intense structure might have acted as a barrier to the release of CUR from the microparticles.

### 3.5 Cell Viability

**Figure 8:**
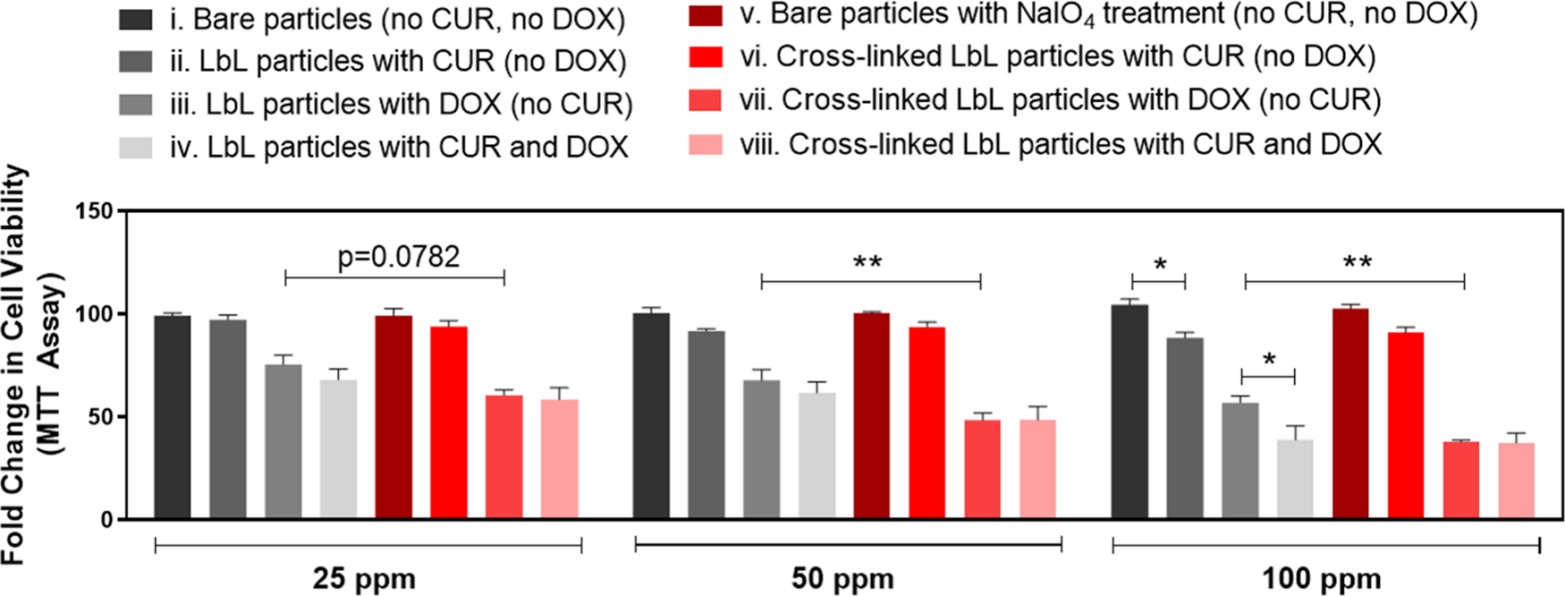
Effect of DOX and CUR-loaded microparticles on the cell viability. Percent fold change in viability of HCT-116 cells upon treatment with (i) bare CaCO_3_ microparticles (no CUR, no DOX), (ii) LbL-coated CUR-containing CaCO_3_ microparticles (no DOX), (iii) DOX postloaded LbL-coated CaCO_3_ microparticles (no CUR), (iv) DOX postloaded LbL-coated CUR-containing CaCO_3_ microparticles, (v) NaIO_4_ treated bare CaCO_3_ microparticles (no CUR, no DOX), (vi) cross-linked LbL-coated CUR-containing CaCO_3_ microparticles (no DOX), (vii) DOX postloaded cross-linked LbL-coated CaCO_3_ microparticles (no CUR), (viii) DOX postloaded cross-linked LbL-coated CUR-containing CaCO_3_ microparticles. The microparticle solutions were prepared at a concentration of 100 ppm and then diluted to 50 and 25 ppm. HCT-116 cells were treated with different concentrations of microparticles for 24 h and then processed for an MTT assay. The average of four biological replicates is shown in the graph.

The viability of HCT-116 cells was determined with different dilutions (25, 50, and 100 ppm) of microparticles loaded with DOX alone, CUR alone, and a combination of DOX and CUR, as well as the bare microparticles (Table 2). Incubation of HCT-116 cells with the bare microparticles showed no significant cytotoxicity at any of the concentrations used (Figure 8). Incubation with varying concentrations of particles loaded solely with DOX led to a dose-dependent reduction in viability at all concentrations, suggesting the successful release of the drug from the particles. Particles loaded with CUR alone showed a modest decrease in viability only at the highest concentration (Figure 8). Additionally, microparticles loaded with a combination of DOX and CUR showed a trend for lower cell viability in a dose-dependent manner compared to DOX alone for all of the particle concentrations tested and reached statistical significance at the highest concentration used (100 ppm).

**Table 2:** Samples for the Determination of Cell Viability Using HCT-116 Cells

sample	description
(i) bare particles	bare CaCO_3_ microparticles (no CUR, no DOX)
(ii) LbL particles with CUR	LbL-coated CUR-containing CaCO_3_ microparticles (only CUR, no DOX)
(iii) LbL particles with DOX	DOX postloaded LbL-coated CaCO_3_ microparticles (only DOX, no CUR)
(iv) LbL particles with CUR and DOX	DOX postloaded LbL-coated CUR-containing CaCO_3_ microparticles (both DOX and CUR)
(v) bare particles with NaIO_4_ treatment	NaIO_4_ treated bare CaCO_3_ microparticles (no CUR, no DOX)
(vi) cross-linked LbL particles with CUR	cross-linked LbL-coated CUR-containing CaCO_3_ microparticles (only CUR, no DOX)
(vii) cross-linked LbL particles with DOX	DOX postloaded cross-linked LbL-coated CaCO_3_ microparticles (only DOX, no CUR)
(viii) cross-linked LbL particles with CUR and DOX	DOX postloaded cross-linked LbL-coated CUR containing CaCO_3_ microparticles (both DOX and CUR)

HCT-116 cells treated with cross-linked particles postloaded with DOX decreased cell viability further when compared to DOX released from the non-cross-linked counterparts at all dilutions (iii versus vii). Cell viability, however, did not decrease any further when HCT-116 cells were treated with DOX postloaded, cross-linked CUR-containing particles (vii versus viii). This is very well corroborated with the high release of DOX, but not CUR, from the cross-linked particles. Thus, the greater release of DOX from the cross-linked particles may have led to a stronger decrease in cell viability; however, since the release of CUR from the cross-linked particles was low, we did not observe any combinatorial effect of the two drugs. We consider two specific scenarios for this observation: (1) The fluorescence intensity of CUR that was released from cross-linked particles was lower than non-cross-linked LbL particles. This could be due to the oxidation of CUR upon treatment with NaIO_4_. Therefore, a partial loss of biological activity of CUR upon oxidation might be the reason for the loss of the combinatorial effect of the two drugs when delivered *via* the cross-linked particles. (2) Cross-linking results in the formation of a more intense film structure, which may make it more challenging for the release of a hydrophobic compound such as CUR. Thus, while cross-linking may have its own advantages, it does not support the release of CUR.

We next determined the CI values for the non-cross-linked LbL microparticles which reflect the degree of interaction of the two drugs (DOX and CUR) and are calculated from the sum of the ratio of the dose of each drug to the dose when used alone when the combination and compound produce 50% efficacy.^62^ The dose–effective curves of each microparticle loaded with DOX or CUR, or a combination of the two, were used to assess synergism, antagonism, or additive effects (Table 3). The DRI and inhibitory effect were calculated from the proliferation data (average MTT assay values from four biological replicates). Our data suggest that the combination of DOX and CUR in the microparticles was synergistic, as the CI values were well below 1. The same analysis, however, could not be carried out with the cross-linked microparticles since a much higher amount of DOX and very low amounts of CUR could be released from those particles.

**Table 3:** Effect of CUR and DOX Loaded LbL-Coated Particles on HCT-116 Cell Viability *In Vitro*

compound		inhibitory effect (%)	parameters				dose reduction index (DRI)	conclusion
CUR (ppm)	DOX (ppm)		*m*	Dm	*r*	CI	CUR/DOX	
25		2.7920		*m*1: 1.09520 ± 0.30565				
50		8.1350	*Dm*1: 571.837					
100		11.5880	*r*1: 0.96319					
	25	24.4610	*m*2: 0.61041 ± 0.03283					
	50	32.2160	Dm2: 162.004					
	100	43.0120	*r*2: 0.99856					
25	25	31.8590	*m*3: 0.87445 ± 0.27457			**0.41558**	42.5014:2.55066	synergistic with favorable dose reduction
50	50	38.1360	Dm3: 67.6221			**0.53505**	27.3534:2.00603	
100	100	61.1110	*r*3: 0.95407			**0.24635**	32.1404:4.64608	

The anticancer and chemosensitizing effects of CUR have been established in several *in vivo* and *in vitro* studies in different types of cancer.^63,64^ However, large doses are often required to exert a killing effect.^65,66^ DOX is an anthracyclin that inhibits a critical enzyme called Topoisomerase-2 that is functional in DNA replication.^67^ DOX is therefore cytotoxic for cells that undergo rapid cell division, such as cancer cells. However, DOX also shows a high cardiotoxicity. Therefore, decreasing the dose of DOX while maintaining its cytotoxicity has been extensively examined. In particular, the combination of DOX and CUR was shown to enhance apoptosis, decrease inflammation, and inhibit metastatic spread in different cancer types more efficiently than DOX alone.^68^ Another study reported that the combination of CUR and DOX in a single nanoparticle formulation could reduce drug resistance by affecting the expression of drug resistance-related genes MDR1 in chronic myeloid leukemia.^69^

The extracellular pH of tumor tissues typically ranges between 6.5 and 7, whereas normal tissues exhibit an extracellular fluid pH of 7.4.^70^ As discussed in Section 3.4.1, LbL microparticles designed in this study could release a greater amount of DOX as the pH was lowered from pH 7.4 to 5.5. Furthermore, higher DOX release at pH 5.5 with cross-linking which might have stemmed from the accumulation of DOX close to the particle surface, may provide an advantage for the preferential release of drugs from a pH-responsive LbL platform in a tumor microenvironment.

CUR was reported to increase the sensitivity of tumor cells to various chemotherapy drugs such as 5-fluorouracil, gemcitabine, and DOX, and reverse drug resistance in colon cancer,^71^ ovarian cancer,^72^ and other cancer types. Our data suggest that a pH-responsive LbL-modified microparticle system can be designed to release CUR and DOX such that the drugs can act in a combinatorial manner to decrease cell viability further than either drug alone. Our data also suggest that although cross-linking might be an effective strategy to target cell viability when used with DOX, it did not provide any advantage for the combinatorial effect of the two drugs.

Finally, it is worth mentioning that we also carried out a cell viability assay using HEK293T cells. These cells, although not malignant, are immortalized. We observed that DOX released from the LbL microparticles could indeed kill HEK293T cells as well (Figure S14). This is not surprising, since many immortalized “normal” cell lines divide very rapidly, including HEK293T cells (doubling time of 24 h^73^). Therefore, DOX, which intercalates into DNA^74^ and activates apoptosis through the inhibition of replication, is very likely to affect the proliferation of HEK293T cells as well. The current study is fundamental in its design. A more refined experimental design in the future with animal studies will provide more reliable data on the effects of the microparticles on normal tissues.

## 4 Conclusions

PiPOX-PEI could be successfully coassembled at the surface with TA using the LbL technique. The driving force for LbL deposition was hydrogen bonding interactions between amide carbonyl groups of PiPOX and phenolic hydroxyl groups of TA as well as electrostatic interactions between protonated amino groups of PEI and phenolate groups of TA. PiPOX-PEI/TA multilayers were cross-linked using NaIO_4_ as the cross-linking agent through the oxidation of phenolic hydroxyl groups of TA to quinone groups by IO_4_^–^ anions, followed by the formation of covalent bonds between the quinone groups and the secondary amine groups of PEI units. Cross-linking of multilayers provided enhanced stability to the multilayers, especially under increasing pH conditions. The potential of PiPOX-PEI and TA multilayer films as drug-releasing platforms was investigated by using DOX and CUR as model anticancer drug molecules. PiPOX-PEI/TA multilayers were deposited onto CUR-containing CaCO_3_ microparticles and postloaded with DOX to generate microparticles for dual drug delivery applications. LbL microparticles displayed enhanced DOX release at moderately acidic conditions due to the disruption of electrostatic interactions between TA and DOX as TA protonated with decreasing pH. On the other hand, increasing the temperature from 25 to 37 °C did not provide a significant difference in the amount of DOX release. This may be attributed to an increase in the critical point of PiPOX upon partial hydrolysis. Besides, the lack of temperature-responsive release at 37 °C may also be due to the low number of PiPOX-PEI layers, which was insufficient to observe a response. Further increasing the temperature to 42 °C enhanced DOX release possibly due to conformational rearrangement within the multilayers and formation of void-like structures, which might have facilitated DOX release. Despite the dissolution of CaCO_3_ microparticles at acidic conditions, CUR release was not responsive to pH variations at 37 °C. This was possibly due to enhanced hydrophobic association between CUR and the layers at increasing temperatures, reducing CUR release from the multilayers. Cross-linking of multilayers enhanced DOX release while reducing the release of CUR. Cross-linked multilayers with relatively intense structures possibly prevented the diffusion of DOX to the inner region and provided accumulation of DOX close to the surface, facilitating its release. The same layers might have abrogated the release of CUR from the particles due to enhanced hydrophobic interactions between CUR and multilayers as the temperature approached the critical point of PiPOX-PEI. The NaIO_4_-induced oxidation of CUR could also account for the relatively low release of CUR from cross-linked microparticles. The release profile of the drugs from non-cross-linked and cross-linked particles corroborated well with the viability of HCT-116 cells treated with the microparticles. Thus, HCT-116 cells treated with non-cross-linked microparticles loaded with both DOX and CUR showed significantly lower cell viability compared to DOX and CUR alone and functioned in a synergistic manner. However, upon cross-linking, the robust release of DOX, but poor release of CUR resulted in a stronger loss of cell viability with DOX alone; however, the combinatorial effect with CUR was lost. This study generated fundamental information about the structure–property relationship in stimuli-responsive LbL films and may form a basis for the design and development of stimuli-responsive drug carrier platforms.

## Supporting Information Available

^1^H NMR spectrum of iPOX, PiPOX, and PiPOX-PEI; GPC spectrum of PiPOX; ATR-FTIR spectrum, XRD pattern, SEM image of CUR-containing CaCO_3_ microparticles, and particle size distribution of CUR containing CaCO_3_ microparticles; particle size distribution of LbL-modified CUR-containing CaCO_3_ microparticles; calibration curves for quantification of CUR loading, DOX loading, and DOX release; DOX release from LbL-coated CUR-containing CaCO_3_ microparticles at 25 °C; fluorescence intensity of CUR released from the CUR-containing CaCO_3_ microparticles, which were exposed to varying concentrations of NaIO_4_ solution; evolution of intensity average size distribution of PiPOX-PEI with increasing temperature; viability of HCT-116 cells treated with DOX, CUR, or their combination; and effect of DOX and CUR-loaded microparticles on cell (HEK293T) viability (PDF)

## Abbreviations

LbL: layer-by-layer
PiPOX-PEI: poly(2-isopropyl-2-oxazoline-*co*-ethyleneimine)
PiPOX: poly(2-isopropyl-2-oxazoline)
TA: tannic acid
DOX: doxorubicin
CUR: curcumin
