# Supplementary material for: Effect of Periodate-Induced Cross-linking on Dual Anticancer Drug Release from Poly(2-isopropyl-2-oxazoline)/Tannic Acid-Based Layer-by-Layer Microparticles
Source: ACS Omega. 2024 Sep 11;9(38):39626–42. doi: 10.1021/acsomega.4c03977 (PMC11425960; doi:10.1021/acsomega.4c03977)
Supplement: Supplementary file 1 — ao4c03977_si_001.pdf [file ao4c03977_si_001.pdf]

Effect of Periodate-induced Cross-linking on Dual Anti-cancer Drug Release from Poly(2-isopropyl-2-oxazoline)/Tannic Acid Based Layer-by-Layer Microparticles

Esma Ugur[a,#], Gökçe Tidim[a,#], Dilara Gundogdu[a,#], Cemre Alemdar[a], Goksu Oral[b], H. Hazal Husnugil[b], Sreeparna Banerjee[b], Irem Erel-Goktepe[a,c,*]

[a]*Department of Chemistry, Middle East Technical University, Ankara, Türkiye*

[b]*Department of Biology, Middle East Technical University, 06800 Cankaya, Ankara, Türkiye*

[c] *Center of Excellence in Biomaterials and Tissue Eng., Middle East Technical University, Ankara, Türkiye*

[*] To whom correspondence should be addressed. Telephone: +90 312 210 3233.

E-mail: erel@metu.edu.tr

[#] Equal contribution

Abbreviations: LbL: layer-by-layer; PiPOX-PEI: poly(2-isopropyl-2-oxazoline-*co*-ethylene imine); PiPOX: poly(2-isopropyl-2-oxazoline); TA: tannic acid; DOX: doxorubicin; CUR: curcumin.

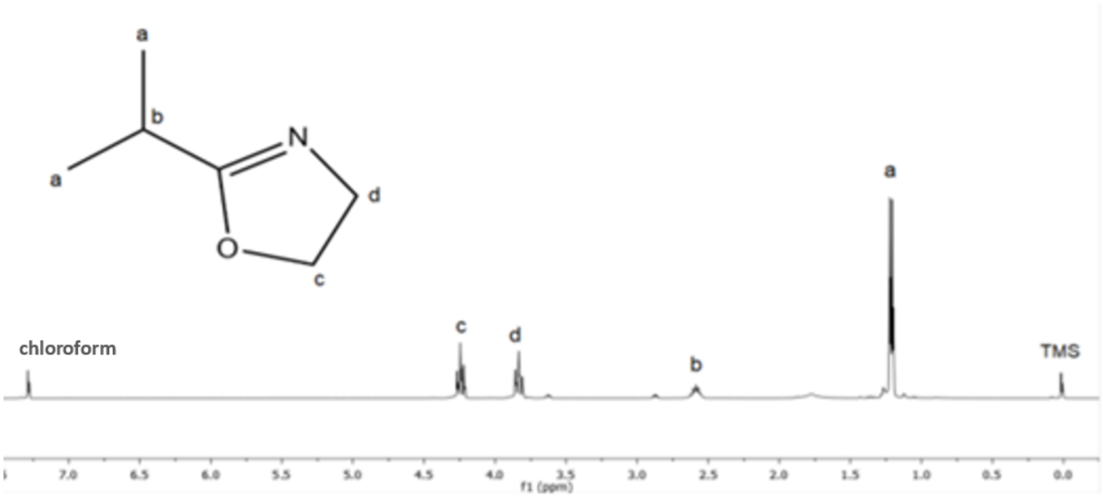

Figure S1. ^{1}H-NMR spectrum of iPOX.

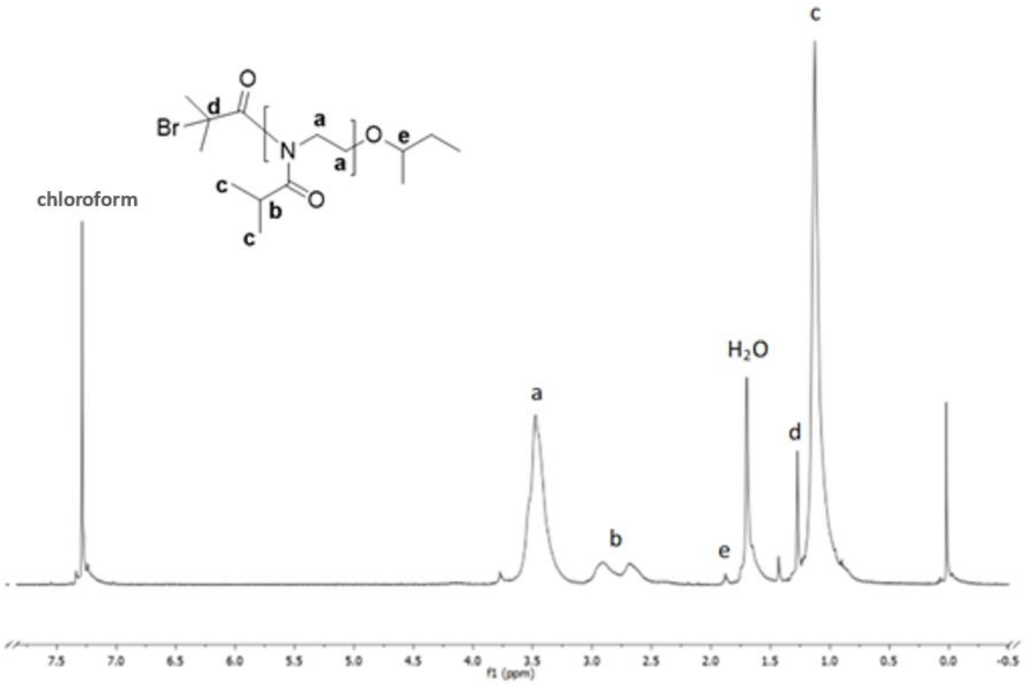

Figure S2. ^{1}H-NMR spectrum of PiPOX.

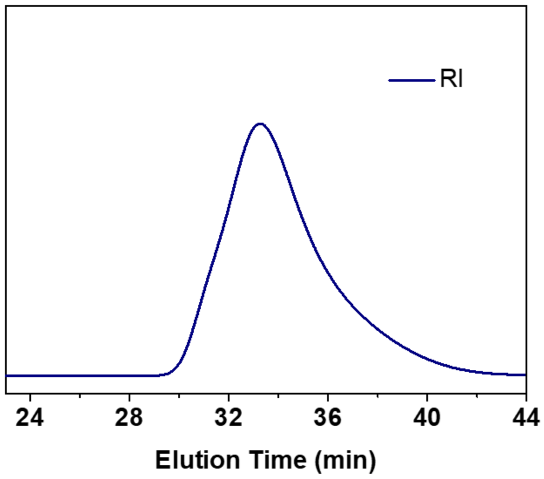

Figure S3. Gel permeation chromatography (GPC) spectrum of PiPOX. Refractive index (RI) detector. M_w = 7429 g/mol, M_n = 6125 g/mol, and polydispersity index, PDI = 1.2.

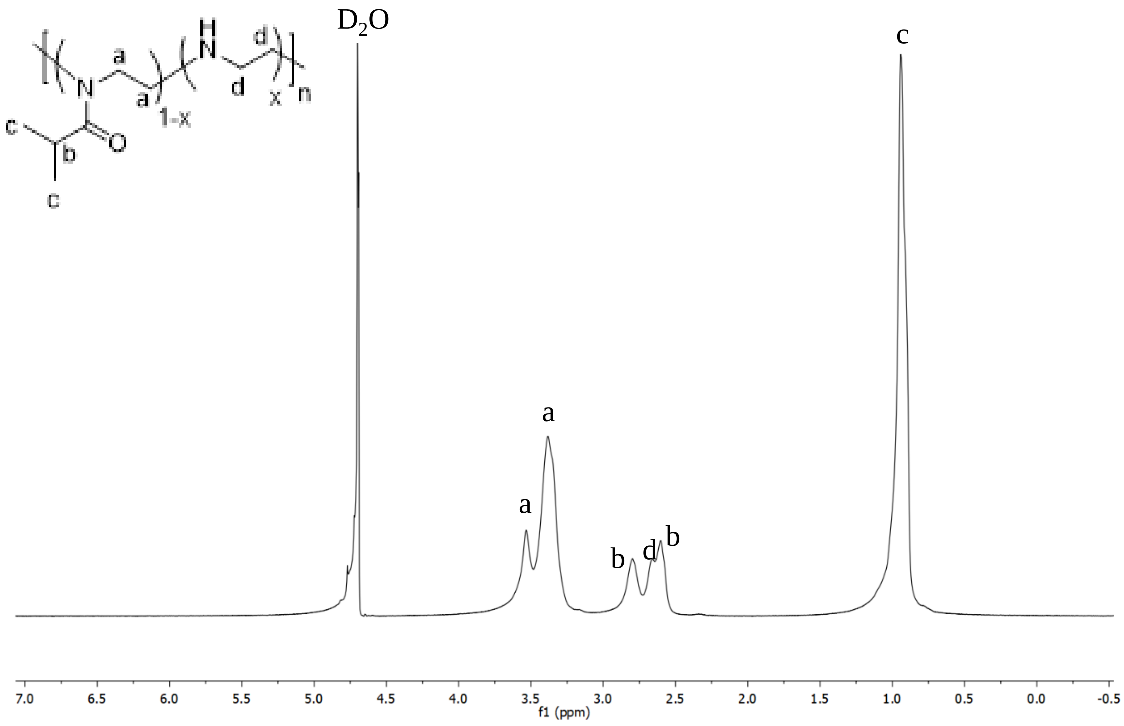

Figure S4. ^{1}H-NMR spectrum of PiPOX-PEI.

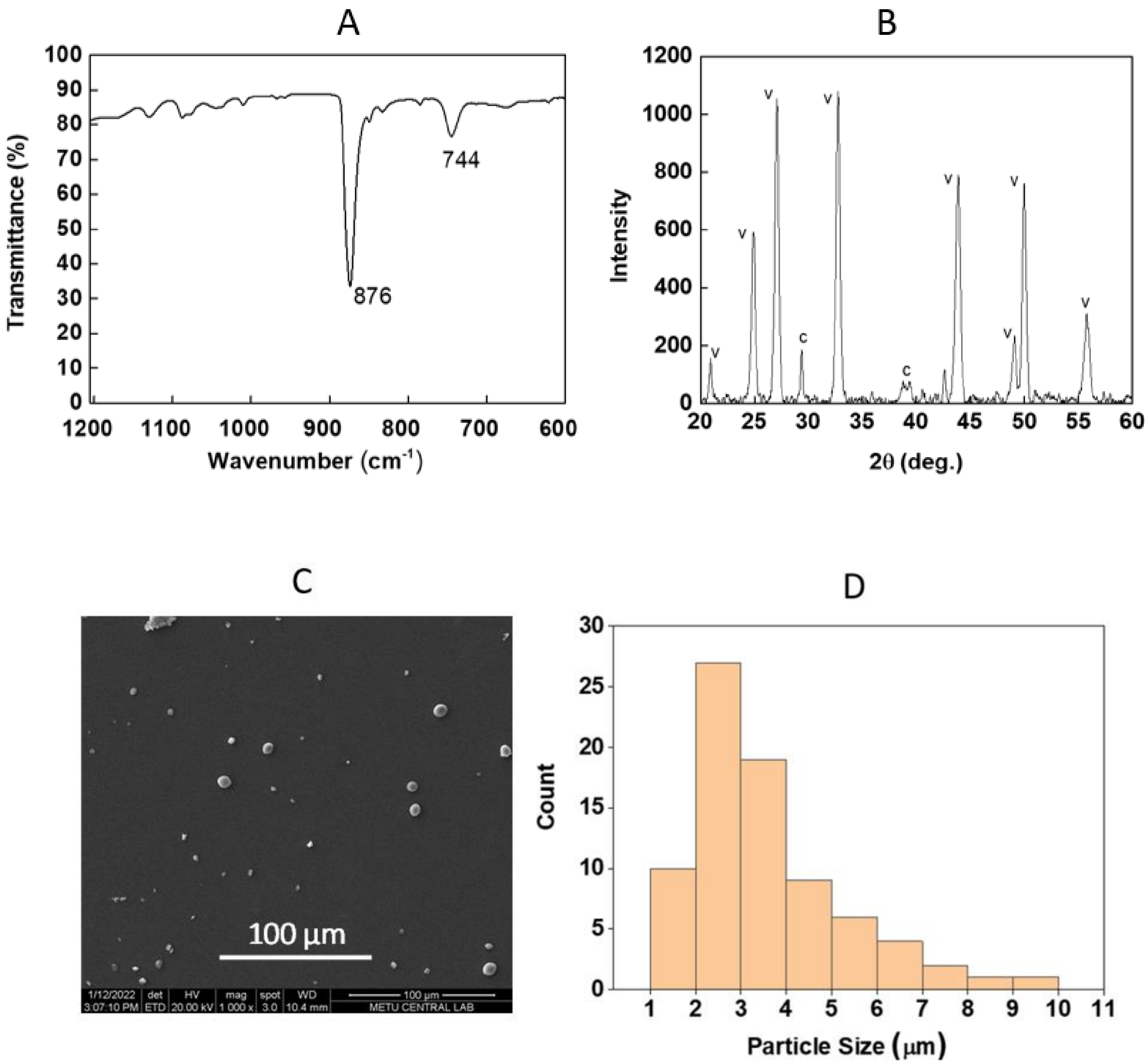

Figure S5. A) ATR-FTIR spectrum of CUR containing $CaCO_3$ microparticles. Absorption bands that are specific to the calcite polymorph of the carbonate ion are observed at 713 cm^{-1}, 848 cm^{-1}, and 1080 cm^{-1}, while the absorption bands of the vaterite form are recorded at 744 cm^{-1}, 876 cm^{-1}, and 1087 cm^{-1}. The peaks which belong to vibrations of CO_3^{2-} ion in vaterite form are found at 744 cm^{-1} and 876 cm^{-1} [1]. **B)** XRD pattern of CUR containing $CaCO_3$ microparticles. Characteristic peaks for vaterite polymorph at 2 Θ = 21.004^o, 24.900^o, 27.047^o, 32.778^o, 42.759^o, 43.848^o, 50.077 ^{o}, 55.805^o, 62.868^o, 71.967^o and 73.593^o as tabulated in PDF 33-0268. **C)** SEM image of CUR containing $CaCO_3$ microparticles. D) Particle size distribution determined from SEM images of CUR containing $CaCO_3$ microparticles.

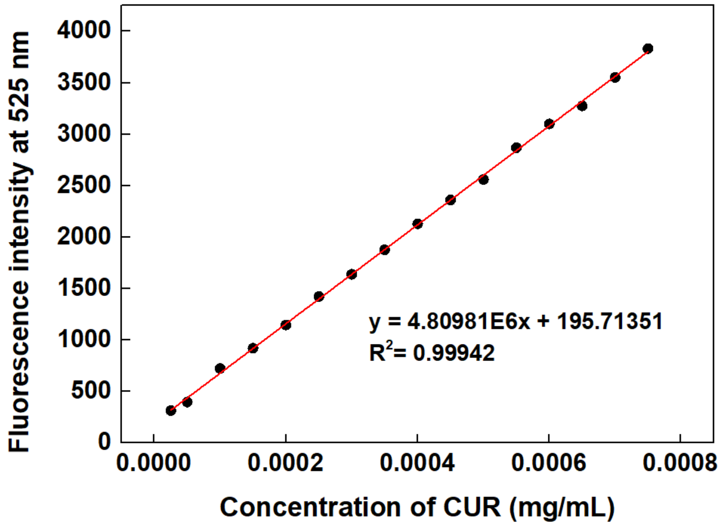

Figure S6. Calibration curve for quantification of CUR in CUR-loaded $CaCO_3$ microparticles in PBS-ethanol mixture (60% ethanol by volume) at pH 5.5.

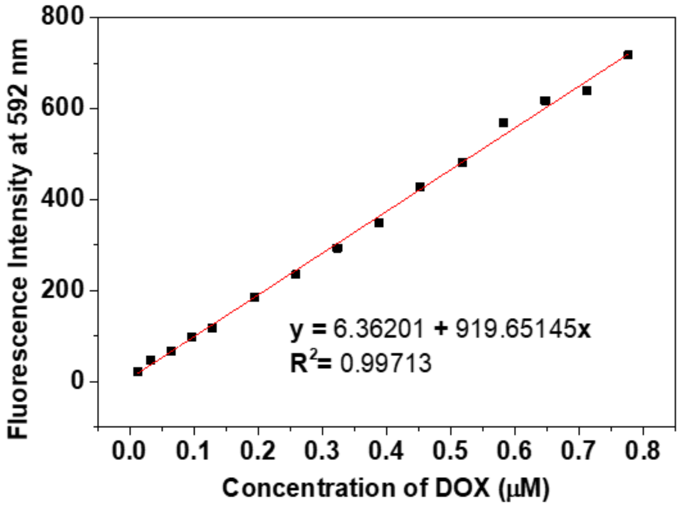

Figure S7. Calibration curves for quantification of DOX loaded into CUR containing $CaCO_3$ microparticles in 10 mM phosphate buffer at pH 7.4

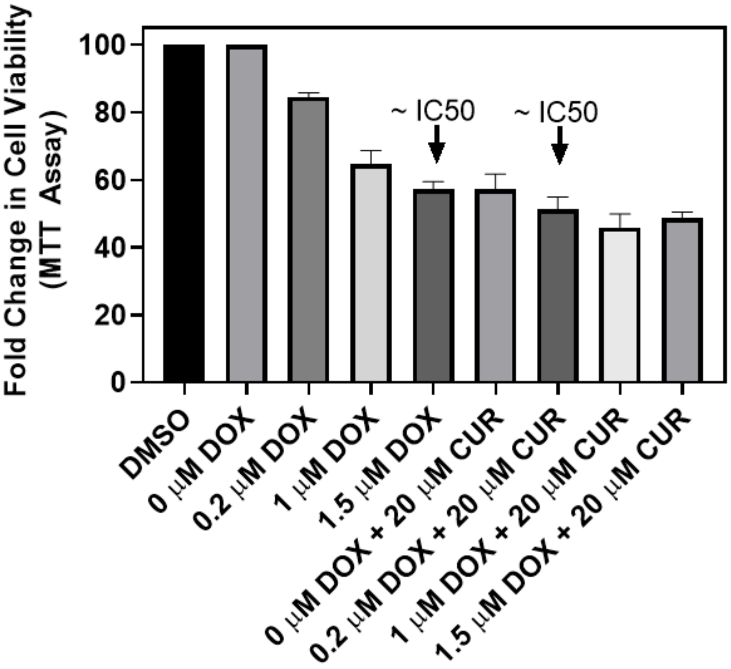

Figure S8. Viability of HCT-116 cells treated with DOX, CUR, or their combination. Cell viability of HCT-116 cells treated with 0, 0.2, 1 and 1.5 µM DOX alone, or in combination with 20µM CUR for 24h. The DMSO concentration (solvent for CUR) was kept at 0.1%. The average of two biological replicates is shown in the graph.

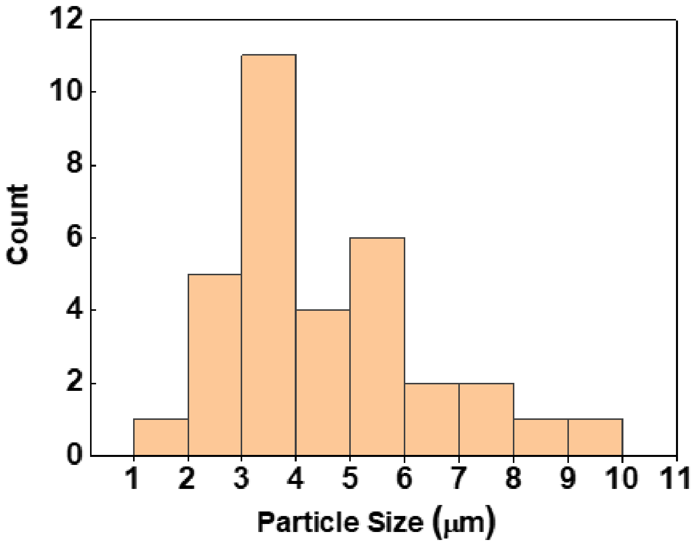

Figure S9. The particle size distribution determined from SEM images of LbL-modified CUR containing $CaCO_3$ microparticles.

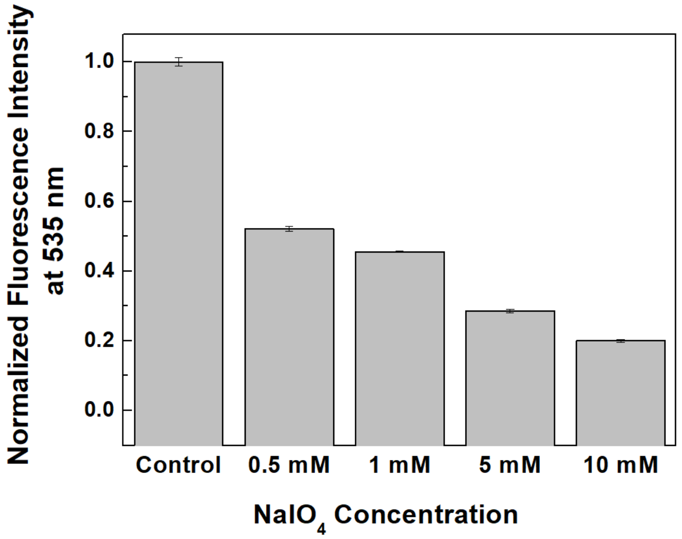

Figure S10. Normalized fluorescence intensity of CUR released from CUR containing $CaCO_3$ microparticles which were exposed to varying concentrations of $NaIO_4$ solution.

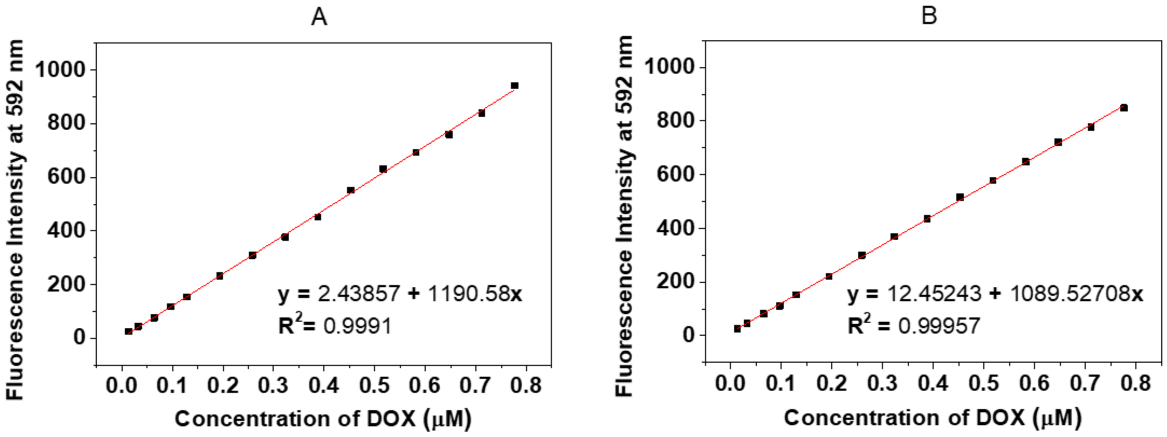

Figure S11. Calibration curves for quantification of DOX release into PBS at (A) pH 5.5 and (B) pH 7.4.

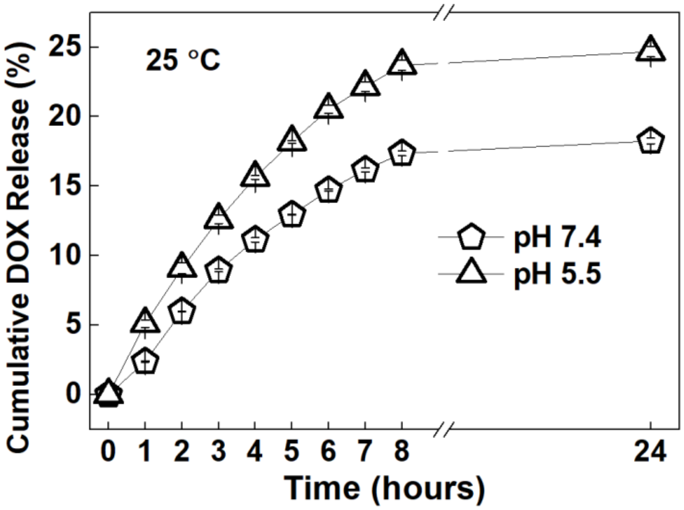

Figure S12. DOX release from TA/PIPOX-PEI coated CUR containing $CaCO_3$ microparticles into PBS at pH 5.5/25 °C and pH 7.4/25 °C.

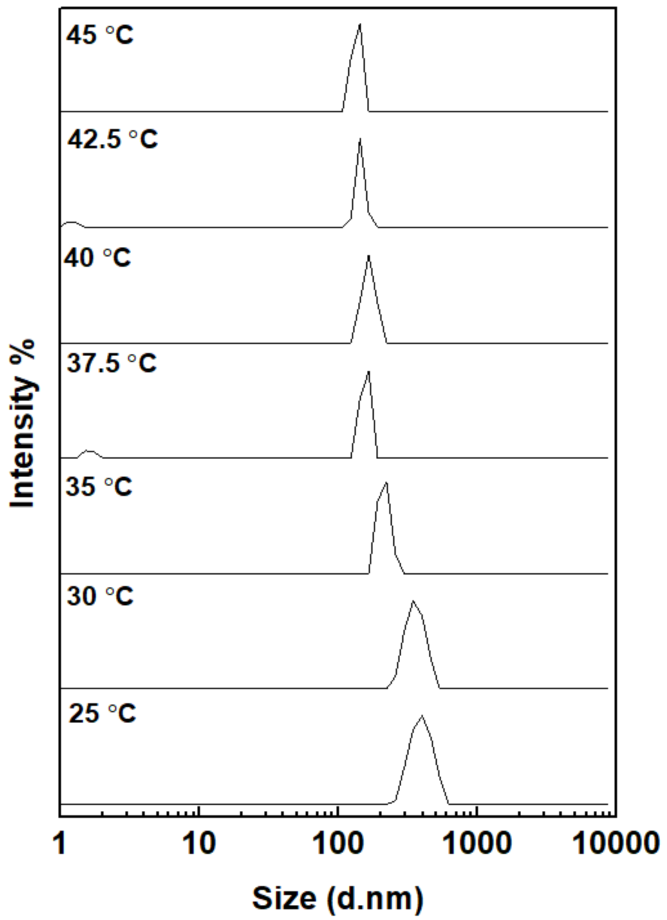

Figure S13. Effect of temperature on aqueous solution behavior of PiPOX-PEI. Evolution of intensity average size distribution with increasing temperature. Hydrodynamic size measurements of PiPOX-PEI (0.5 mg/mL, prepared in DI water) were performed at varying temperatures with Zetasizer Nano-ZS equipment. During heating of the polymer solution, temperature was increased by 5 °C between 25 °C and 30 °C and by 2.5 °C between 35 and 45 °C.

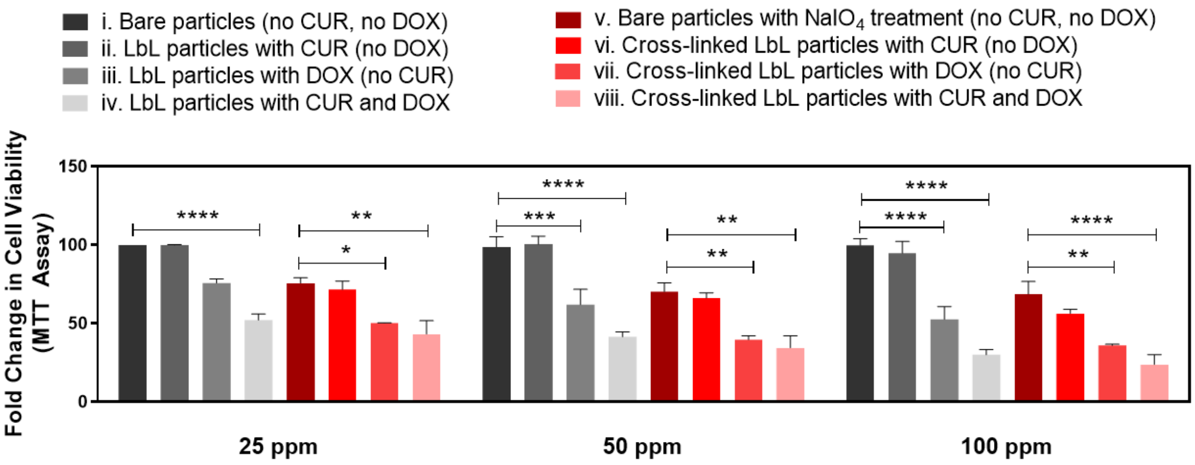

Figure S14. Effect of DOX and CUR loaded microparticles on cell viability of the non-tumorigenic cell line HEK293T. Percent fold change in cell viability upon treatment with i: bare $CaCO_3$ microparticles (no CUR, no DOX), ii: LbL-coated CUR containing $CaCO_3$ microparticles (no DOX), iii: DOX post-loaded LbL-coated $CaCO_3$ microparticles (no CUR), iv: DOX post-loaded LbL-coated CUR containing $CaCO_3$ microparticles, v: $NaIO_4$ treated bare $CaCO_3$ microparticles (no CUR, no DOX), vi: Cross-linked LbL-coated CUR containing $CaCO_3$ microparticles (no DOX), vii: DOX post-loaded cross-linked LbL-coated $CaCO_3$ microparticles (no CUR), viii: DOX post-loaded cross-linked LbL-coated CUR containing $CaCO_3$ microparticles. The microparticle solutions were prepared at a concentration of 100 ppm and then diluted to 50 and 25 ppm. HEK293T cells were treated with different concentrations of microparticles for 24h and then processed for an MTT assay. The average of two biological replicates is shown in the graph.

References

[1] J. Chen, L. Xiang, Controllable synthesis of calcium carbonate polymorphs at different temperatures, Powder Technol. 189 (2009) 64–69. https://doi.org/https://doi.org/10.1016/j.powtec.2008.06.004.
